# MD-SeeGH: a platform for integrative analysis of multi-dimensional genomic data

**DOI:** 10.1186/1471-2105-9-243

**Published:** 2008-05-20

**Authors:** Bryan Chi, Ronald J deLeeuw, Bradley P Coe, Raymond T Ng, Calum MacAulay, Wan L Lam

**Affiliations:** 1 Department of Cancer Genetics and Developmental Biology, British Columbia Cancer Research Centre, Vancouver, BC, Canada; 2 Department of Computer Science, University of British Columbia, Vancouver, BC, Canada; 3 Department of Cancer Imaging, British Columbia Cancer Research Centre, Vancouver, BC, Canada

## Abstract

**Background:**

Recent advances in global genomic profiling methodologies have enabled multi-dimensional characterization of biological systems. Complete analysis of these genomic profiles require an in depth look at parallel profiles of segmental DNA copy number status, DNA methylation state, single nucleotide polymorphisms, as well as gene expression profiles. Due to the differences in data types it is difficult to conduct parallel analysis of multiple datasets from diverse platforms.

**Results:**

To address this issue, we have developed an integrative genomic analysis platform MD-SeeGH, a software tool that allows users to rapidly and directly analyze genomic datasets spanning multiple genomic experiments. With MD-SeeGH, users have the flexibility to easily update datasets in accordance with new genomic builds, make a quality assessment of data using the filtering features, and identify genetic alterations within single or across multiple experiments. Multiple sample analysis in MD-SeeGH allows users to compare profiles from many experiments alongside tracks containing detailed localized gene information, microRNA, CpG islands, and copy number variations.

**Conclusion:**

MD-SeeGH is a new platform for the integrative analysis of diverse microarray data, facilitating multiple profile analyses and group comparisons.

## Background

**Figure 1:**
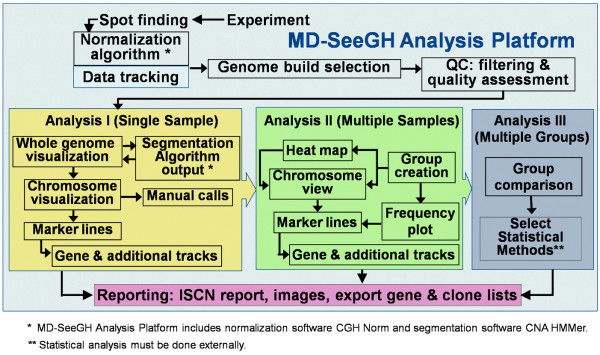
Flow Diagram Summarizing the MD-SeeGH Platform.

Recent advances in global genomic profiling methodologies have enabled multi-dimensional characterization of biological systems. The deciphering of downstream effects of genetic and epigenetic alterations on expression patterns is paramount in understanding disease phenotype and requires the integration of segmental DNA copy number status, DNA methylation state and single nucleotide polymorphism (SNP) status. The large scale generation of such data has created a need for robust software to integrate multiple large genetically linked data sets generated on diverse microarray platforms. Although several visualization software programs are available publicly (for example [1-5]), there is a growing demand for new bioinformatics tools that allow for the concerted analysis of multiple genome-wide experiments derived from different experimental platforms [6]. Blue Fuse [7] and CGH Analytics [8], two commercially available software tools, offer integrative analysis with expression data but neither contain the full feature set that we deem necessary (Table 1). SeeGH (v1.6) was initially developed to view primarily array CGH data [2] but as we continued to use and develop the software we realized that there was a need for the combined analysis of multi-platform data which required significant upgrades to the initial version of SeeGH. Here we present MultiDimensional-SeeGH (MD-SeeGH) analysis platform, a powerful software tool that allows users to quickly and easily analyze genomic anchored datasets comprised from multiple genomic experiments (Figure 1).

**Table 1:** Feature comparison of integrative analysis platforms

	MD-SeeGH	VAMP/CAPweb	ISACGH	CGH Analytics	CGHPRO	CGH Explorer	Blue Fuse	ArrayCyGHt	M-CGH	SeeGHv1.6
Segmentation	✓	✓	✓	✓	✓	✓	✓		✓	
Normalization	✓	✓	✓		✓		✓	✓	✓	
ISCN Reporting	✓						✓			
Integrative Analysis with Expression data	✓	✓	✓	✓			✓			
Multiple Sample Visualization	✓	✓	✓	✓	✓	✓				
Gene Tracks	✓	✓	✓	✓		✓				
Other Tracks (ie.miRNA, CNV, CpG island, etc.)	✓	✓								
Links to external websites (ie. NCBI, UCSC, etc.)	✓	✓	✓	✓		✓		✓		
Mapping files (different Genomic Builds)	✓		✓	✓						
Integration of third party analysis tools	✓		✓	✓	✓					
Frequency Plot	✓	✓	✓	✓	✓	✓				
Heatmap	✓	✓		✓		✓				
Group analysis	✓	✓	✓							
Free public access	✓	✓	✓		✓	✓		✓		✓
Data storage (samples only loaded once)	✓	✓			✓					
Web based software		✓	✓					✓		
References		[6, 20]	[21]	[8]	[1]	[3]	[7]	[22]	[5]	[2]

## Implementation

MD-SeeGH was created using Borland's C++ Builder6 development platform. MySQL is used as the backend database server which is freely available for download. The MD-SeeGH software was developed and tested on Windows XP and Vista. The software and documentation are publicly available online [9].

From our interaction with researchers and clinicians, we note that some of the key features required by integrative analysis software for handling diverse genomic datasets are: (1) flexibility (2) data quality assessment, (3) visualization (4) single and multiple sample analyses, (5) multi group analyses, and (6) comprehensive reporting. To highlight how MD-SeeGH performs these functions, we discuss the parallel analysis of genomic and epigenomic array comparative genomic hybridization (CGH) data as well as the analysis of multidimensional data sets including gene expression, comparative genomic hybridization, differential methylation, and single nucleotide polymorphisms.

## Results and discussion

The following sections describe the flow chart summarizing the functional modules of MD-SeeGH (Figure 1).

### Data tracking, preprocessing and import of data

Microarray data captured after hybridization, scanning, and spot finding are imported into MD-SeeGH as tab-delimited text files. At this time each dataset can be annotated to facilitate data tracking, easy recall, and group definition. Clinical information can also be entered and associated with each dataset. Microarray image data are commonly normalized to remove intensity and spatial biases. For example, the output of a stepwise normalization algorithm, CGH-Norm, is seamlessly imported into MD-SeeGH [10].

### Flexible genome mapping and annotation

To relate array spot information to a specific genomic map location, it is important to use the appropriate genome build (e.g. UCSC Human Genome Freeze Mar 2006/hg18). We have embedded the genomic locations of array features within MD-SeeGH for all available genome builds utilized by the common genomic microarray platforms. New mapping information (future builds) can be easily imported into MD-SeeGH using tab-delimited text files containing base pair information for each array feature. This provides the user with the ultimate flexibility of remapping entire datasets against any genomic build without having to manually transform each individual dataset and reload them into MD-SeeGH [see Additional file 1].

### Quality assessment of data

MD-SeeGH allows filtering of spot data based on standard deviation of replicate measurements and spot signal to noise ratios based upon user-defined parameters. The percentage of spots discarded by filtering and the average signal to noise ratio are displayed for each experimental dataset. A recently described phenomenon in array CGH experimentation (regardless of array platform used) has been the identification of a recurrent artefact pattern that is independent of the copy number status [11,12]. We have created a tool to measure and compensate for this identified recurrent baseline pattern (noise) within array CGH experiments [11] [see Additional file 1].

### Detection of genetic alterations

**Figure 2:**
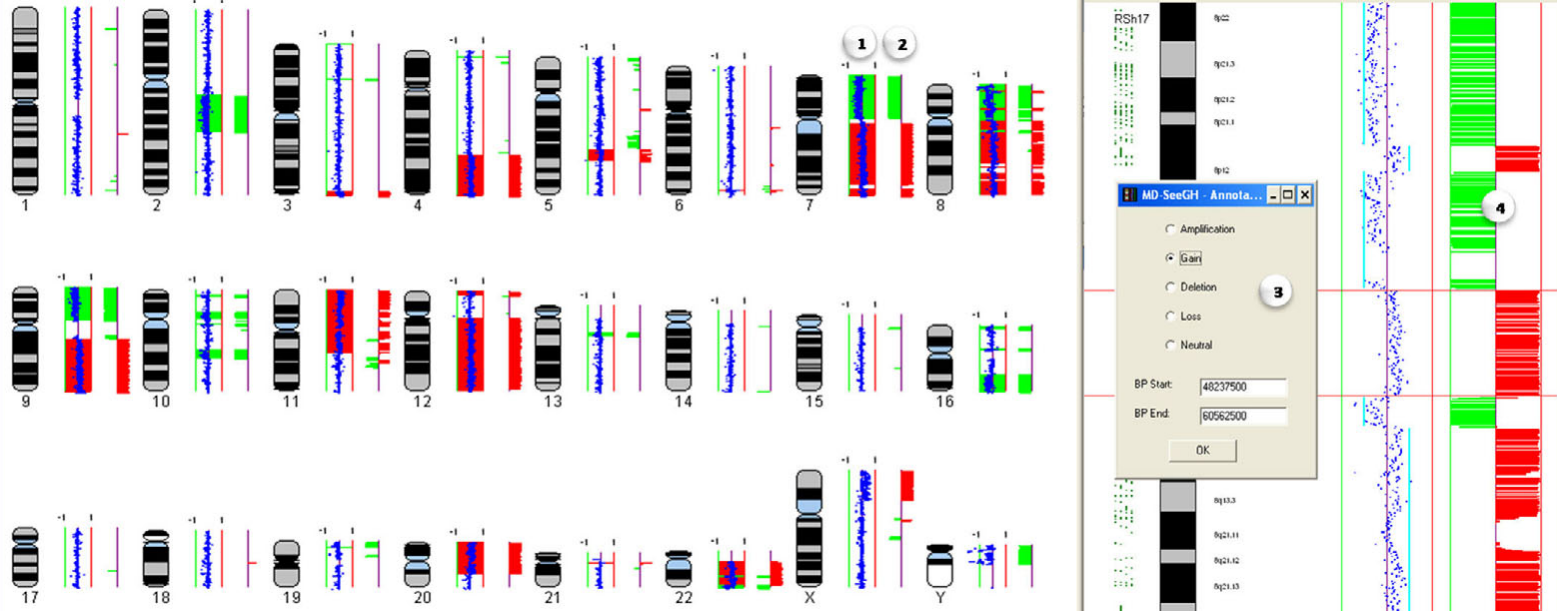
**Identification and Annotation of Altered Regions**. The Annotate Regions option is an analysis tool that allows you to record regions of interest (i.e. amplifications, deletions), save them to the database, and create ISCN reports. Annotating regions can be used side by side with segmentation probabilities to verify the called regions and can also be used to compare amplification and deletions across multiple samples or create Frequency Plots. Numbers indicate genomic view of (1) annotated regions and (2) segmentation calls, and chromosome view of (3) annotation form where user can mark the region as an amplification, gain, deletion, loss or neutral region and (4) segmentation calls which aid in making the calls.

Once the imported data has undergone the appropriate quality assessment, analysis can begin with the identification of alterations for each sample (Analysis I – Figure 1). Many segmentation algorithms have been developed to identify regions of alteration, each with their unique strengths/weaknesses[13]. Given that each microarray platform may require a specific segmentation algorithm, for example a modified Hidden Markov Model for segmentation of array CGH data [14]. MD-SeeGH allows the user to import the output from such algorithms as CNA HMMer, DNAcopy and aCGH Smooth [14-16]. (Can also import any dataset where each spot is annotated with a call). The result of each segmentation output is displayed beside each measured experimental data feature to assist the user in assigning copy number representation to the data within an experiment (Figure 2) [see Additional file 1].

### Gene and additional tracks

**Figure 3:**
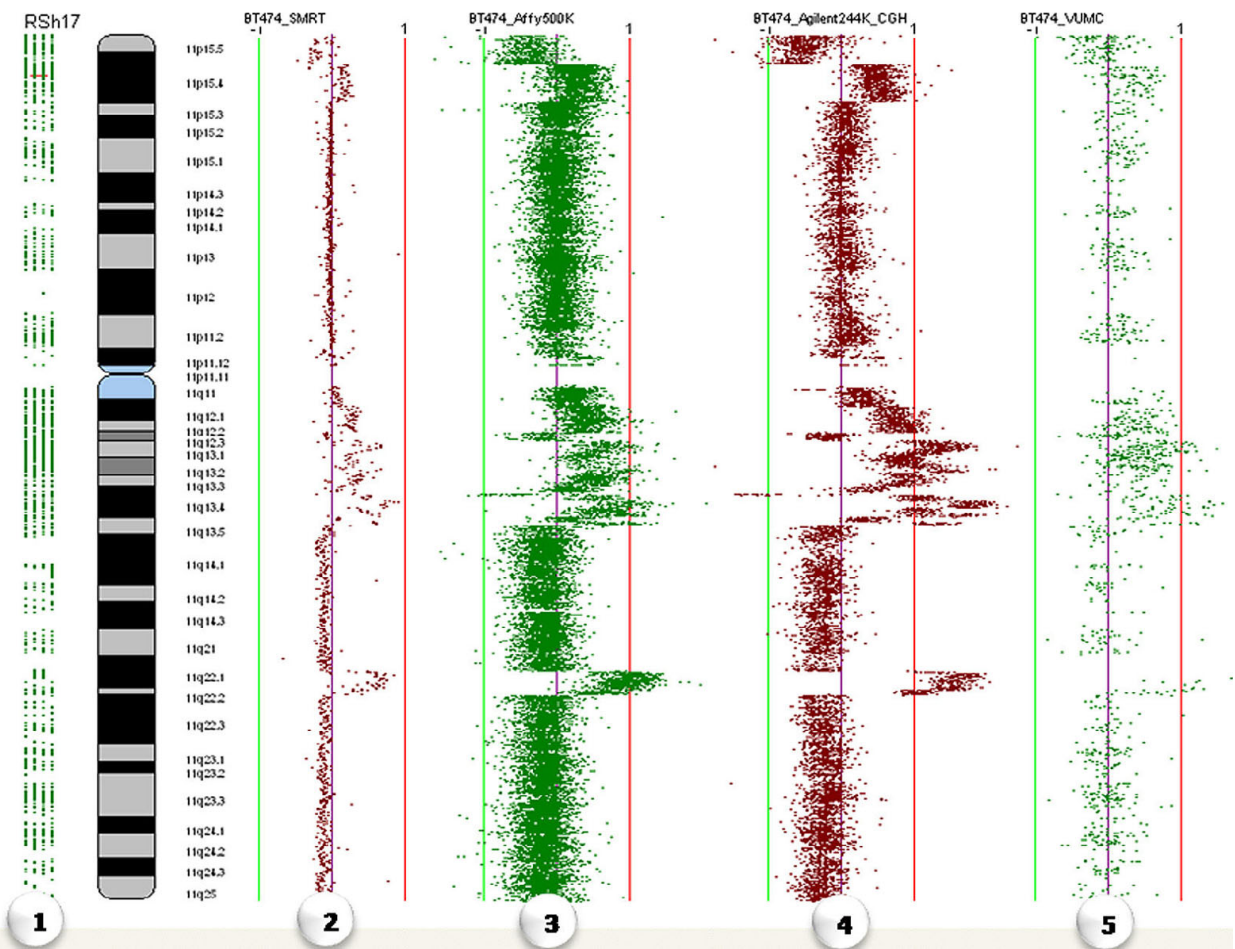
**Multiple Analyses of Different Platforms**. Comparison of the same sample (BT474 cell line) across the following different array platforms: (1) RefSeq GeneTrack, (2) SMRT array, (3) Affymetrix GeneChip human mapping 500 K set, (4) Agilent 244A, and (5) VUMC MACF human 30 K.

Accessible information embedded in MD-SeeGH includes all annotated RefSeq genes, microRNAs[17], CpG islands[18], and natural copy number variations (CNVs) [19]. However, we provide the user with the flexibility to display any genomic annotated fields as a track beside the experimental data (Figure 3a). Data within gene tracks can be selected to display information about each gene from multiple sources (OMIM, Entrez mRNA, Entrez protein, Pubmed, and UCSC genome browser). The gene and additional tracks allow the user to determine if a specific spot on their array overlaps with a specific gene, microRNA, CNV, etc. Up to 4 tracks are visible at all zoom levels during analysis and visualization. Alternately, an entire region can be displayed via the UCSC genome browser at the touch of a button [see Additional file 1].

### Multiple sample analyses

**Figure 4:**
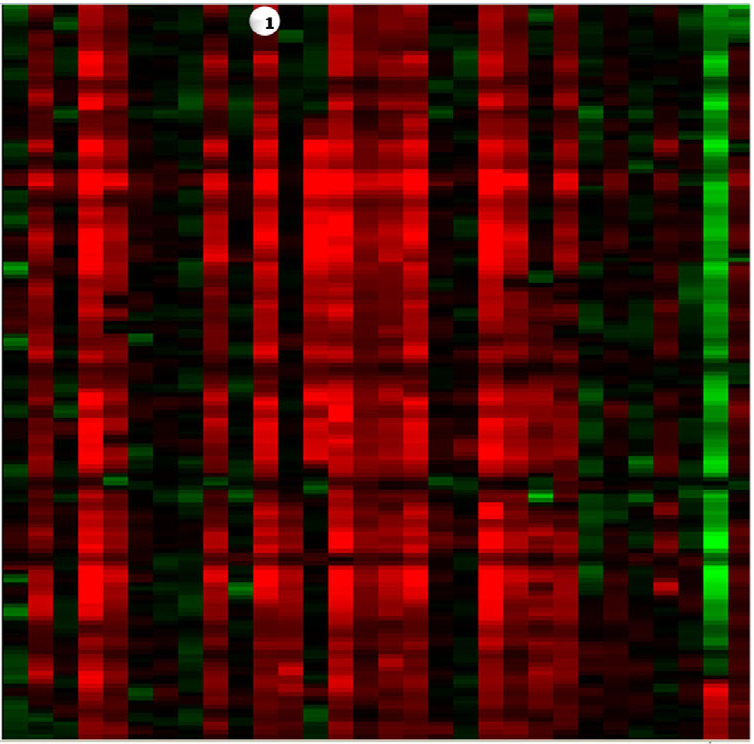
**Heatmap for Defining Recurring Features**. Heatmap allows the user to analyze up to 100 samples and find common regions of amplification or deletion across groups of samples. (1) Each column represents a moving average heatmap of a single sample. Amplifications are shaded red and deletions are shaded green. The greater the moving average ratio the brighter the red (-) or green(+).

**Figure 5:**
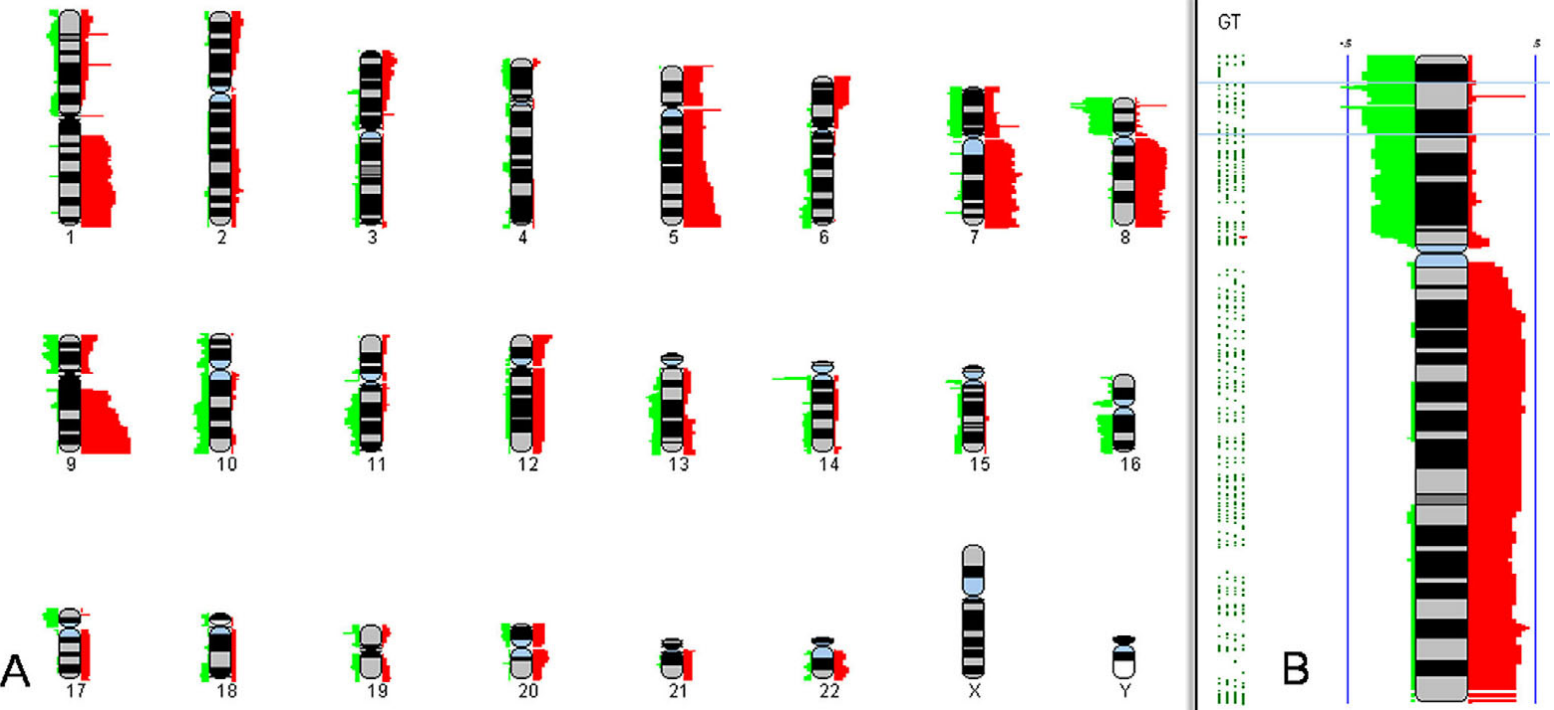
**Frequency Plot**. The Frequency Plot can be used to analyze a group of samples and find minimal regions of amplifications or deletions. Frequency plot scoring for up to 1000 samples can be created within SeeGH or created externally and loaded into SeeGH. Once loaded each sample is stored in the SeeGH database. Amplifications are shaded red and deletions are shaded green. Left panel (A) shows genome view and right panel (B) shows the chromosome view.

In MD-SeeGH, data from up to 50 experiments can be aligned for direct comparison allowing for cross platform analysis or viewing multiple patient samples from the same disease type (Analysis II – Figure 1) (Figure 3). Multiple samples are viewed one chromosome at a time and can be easily changed via a chromosome drop-down box. There is no limit on the density of arrays when viewing multiple samples. Of course the larger the arrays the longer it takes to load. During testing of the Multiple Alignment feature we were able to load 50 Agilent 244 k arrays in 1 minute and 20 seconds on a computer with 2 GB of RAM and a 2.7 Ghz processor. On the same machine, 50 SMRT 32 K arrays took less than 15 seconds to load. In addition, up to 100 experiments can be analyzed and summarized as a heatmap (Figure 4). The heatmap is generated by calculating a moving average across each experiment and allows for a quick way to find regions of interest across a large number of experiments. A given region of interest identified on the heatmap can be further investigated in detail by directly switching to multiple alignment of individual profiles. MD-SeeGH also has the ability to analyze up to 1000 samples as a frequency plot showing percent of samples altered (Figure 5). The frequency plot feature gives the user the ability to identify minimal regions of interest across large datasets. Frequency plots can be created within MD-SeeGH for any datasets from the same array platform that have called data. The frequency of alteration is calculated for each spot of the selected array platform. When creating frequency plots within MD-SeeGH, the maximum density allowed is 25,000 spots per chromosome for a total of 600 K spots in the array. Frequency plot data can also be created externally and loaded into MD-SeeGH. When analysing frequency plots, whole genome and individual chromosome plots are available to the user [see Additional file 1].

### Platform independence and integrative analyses of multi-dimensional datasets

**Figure 6:**
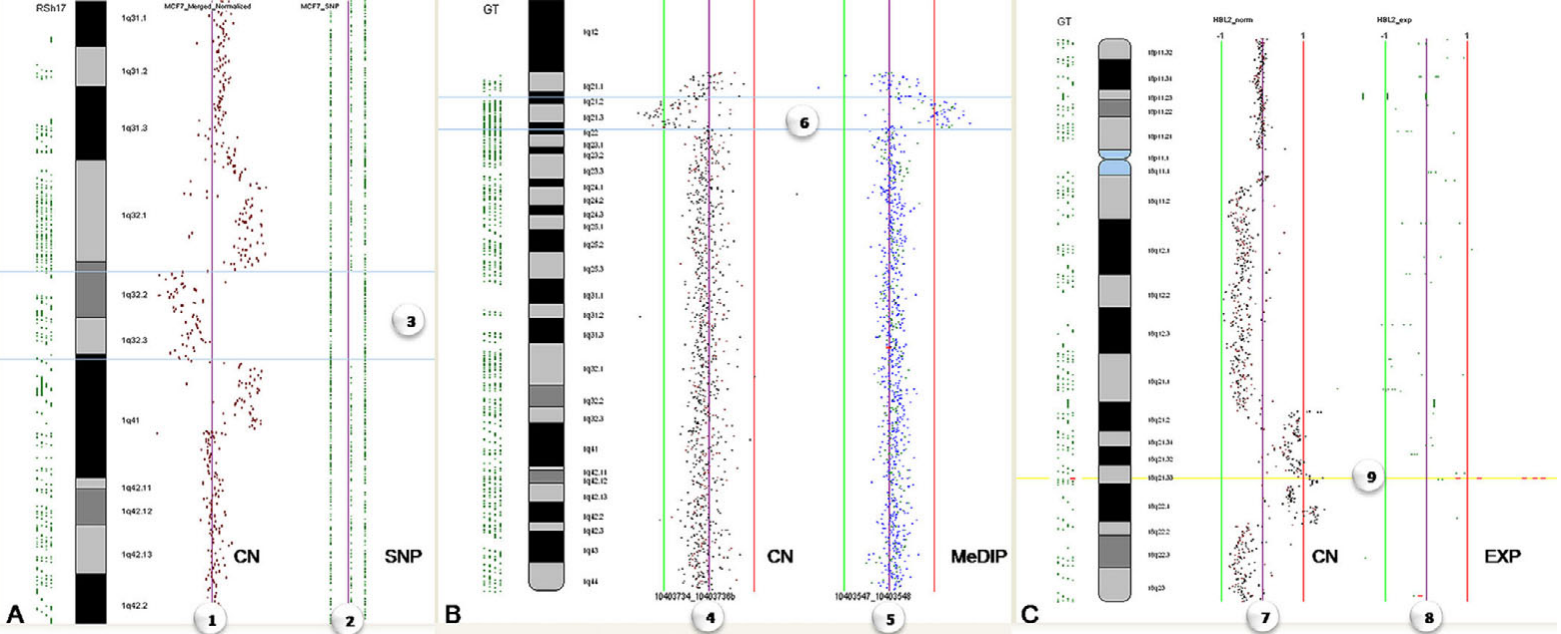
**Analysis of Multi-Dimensional Data: Integration of Different Types of Data**. a. Integration of copy number (CN) data in the context of SNP profile for MCF7 cells. (1) SMRT array CGH profile displayed alongside (2) Affymetrix SNP array – Homozygous AA on left, Heterozygous AB in middle, Homozygous BB on right. Region between the blue lines (3) shows a copy number loss (left) on chromosome 1 associated with LOH (right). b. Integration of epigenetic and genomic profiles. (4) Methylated DNA immunoprecipitation (MeDIP) array CGH profile displayed alongside a (5) SMRT array CGH. Region between the blue lines (6) shows both hypomethylation (left) and copy number change (right). c. Integration of Array CGH and Lymphochip cDNA Gene Expression. (7) SMRT array CGH profile displayed alongside a (8) cDNA expression (EXP) profile (Lymphochip). Yellow highlighted region (9) shows a BCL2 gain and overexpression.

Any data that is tied to a genomic base pair position can be loaded into MD-SeeGH. This includes single channel Affymetrix SNP arrays and double channel Agilent, Nimblegen, Illumina, and SMRT arrays. Within multiple sample analyses, it is not a requirement that all data be created from the same microarray platform. This capability can be utilized to assess the differing characteristics of microarray platforms (Figure 3) or combine data derived from the latest platforms with data created using older platforms. This functionality is increasingly desirable to analyze multi-dimensional datasets, for example, the integration of methylation patterns, copy number alterations and single nucleotide polymorphisms (Figure 6a,b). However, its main advantage is in analyzing gene expression changes in the context of these genetic features (Figure 6c).

### Comparative analysis of multiple groups

**Figure 7:**
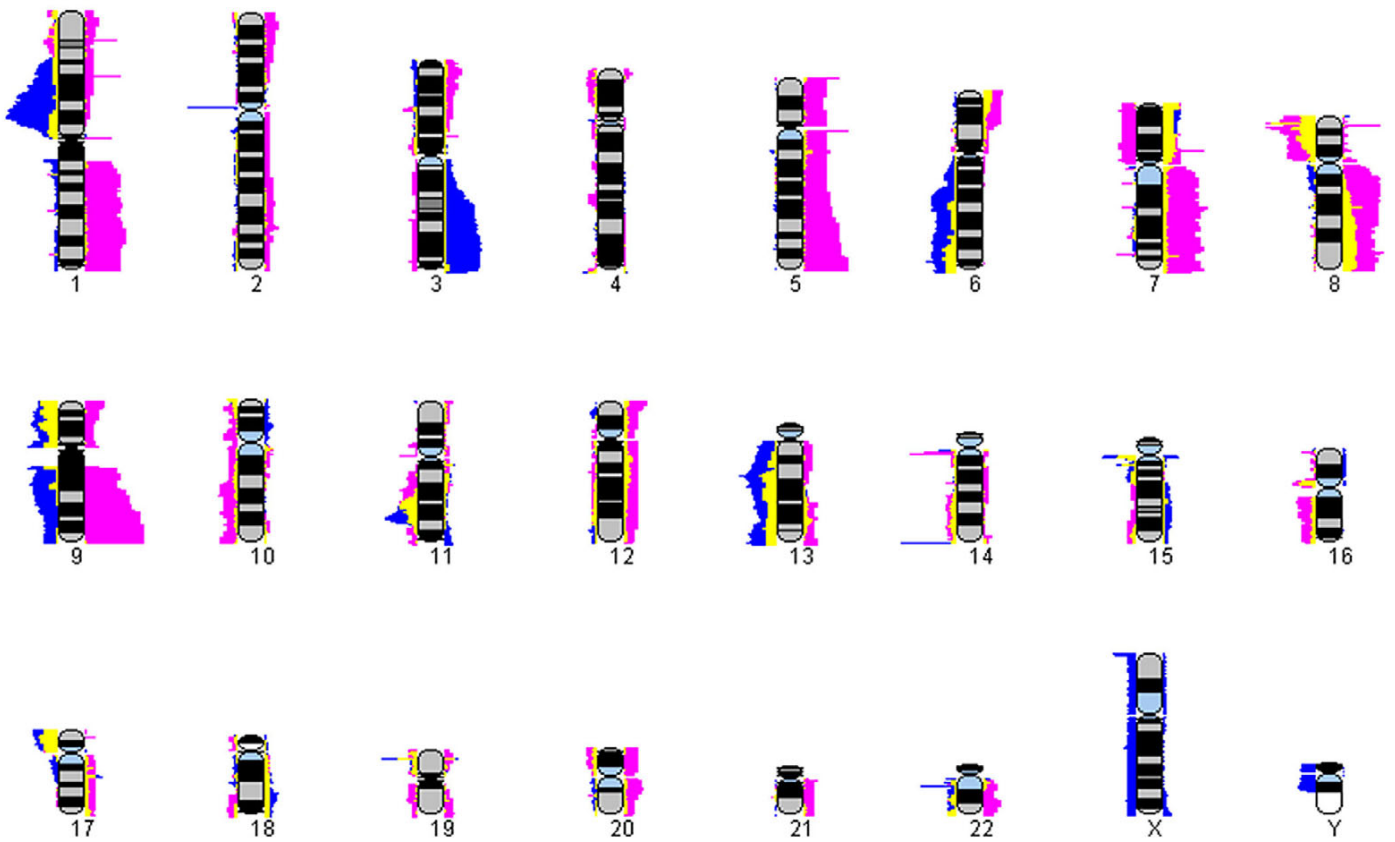
**Group Comparison**. Frequency plot comparison of two different groups (derived from two frequency plot datasets) representing different disease types. Once frequency plots have been loaded/created in SeeGH the user can compare two frequency plots using the overlay feature. Each group is a different color (Group 1 – Fuschia, Group 2 – Blue) and any overlapping regions are a third color (Intersection – Yellow). This is a useful feature to determine similarities and differences between two groups of samples.

An additional level of analysis is the ability to compare two groups of experiments to identify differences between them. In MD-SeeGH this is achieved through the comparison of frequency plots (Figure 7). Permutation testing, Fisher's exact tests, and other statistical tests can be easily conducted using data exported from MD-SeeGH. These statistical analyses provide p-values for the differences between the two groups [see Additional file 1].

### Exporting results and analysis reporting

**Figure 8:**
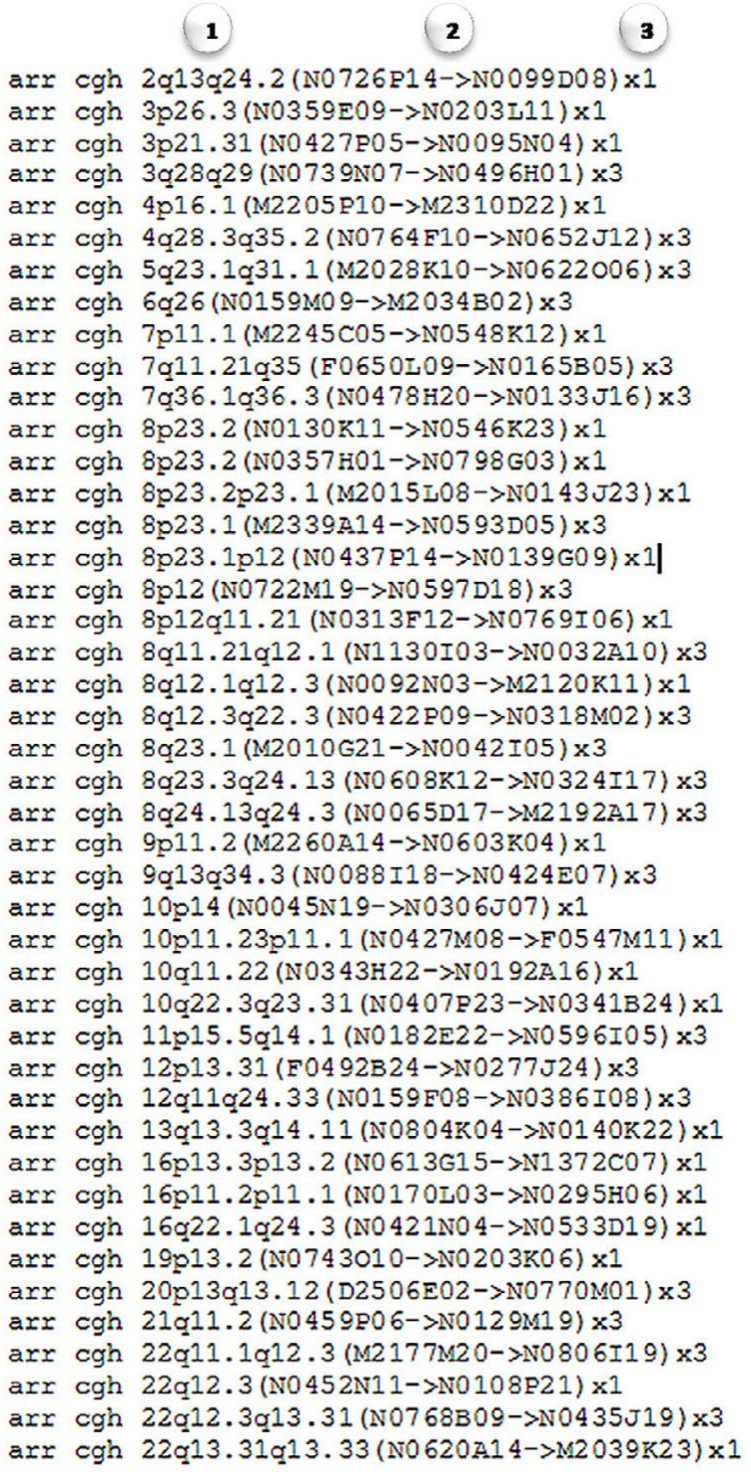
**Sample ISCN report exported from MD-SeeGH**. Tab-delimited ISCN report exported from MD-SeeGH. Regions are noted with (1) chromosome banding position, (2) first and last clone/feature of the region, and (3) whether it is an amplification or deletion. Amplifications or copy number gain is marked as 'x3', while deletions or copy number loss is marked as 'x1'.

MD-SeeGH provides three main ways to export data. Firstly, any image can be saved as either a jpeg or a bitmap file. Secondly, noting that new analysis algorithms are constantly being developed, we built in the ability to export data from MD-SeeGH in a tab-delimited text format that can be readily manipulated with other software/statistical packages. Finally, in a clinical or repetitive standardized analysis setting, the attachment of an entire array CGH data file to a report is unrealistic; therefore, we allow direct generation of a cytogenetic report, formatted with the latest ISCN standard for array experiments (Figure 8) [see Additional file 1].

## Conclusion

In conclusion, we have developed a new platform for the integrative analysis of diverse microarray data, facilitating multiple profile analyses and group comparisons.

## Availability and requirements

**Project name: **MD-SeeGH

**Project home page:**

**Operating system: **Microsoft Windows XP, Microsoft Windows Vista

**Programming language: **C++, SQL

**Other requirements: **MySQL database

**License: **Academic software license must be agreed upon during installation.

**Any restrictions to use by non-academics: **Yes

## Authors' contributions

BC was the principle programmer of the source code. RJdL, BPC and RTN contributed ideas for software features and requirements. CM and WLL are principle investigators of this work. All authors contributed to writing the manuscript. All authors read and approved the final manuscript.

## Supplementary Material

Additional file 1 Supplementary figures Click here for file
