# Supplementary material for: MD-SeeGH: a platform for integrative analysis of multi-dimensional genomic data
Source: BMC Bioinformatics. 2008 May 20;9:243. doi: 10.1186/1471-2105-9-243 (PMC2408605; doi:10.1186/1471-2105-9-243)
Supplement: Additional file 1 — Supplementary figures [file 1471-2105-9-243-S1.pdf]

**Figure S1. Allows Analysis of Different Genomic Builds and Platforms**

The Mappings/Tracks/Chromosomes tab is used to load new mapping information along with corresponding chromosome information and tracks. This allows one to add mapping files for new genomic builds when they are released as well as new platforms. This feature is essential to allow for any genomically anchored platforms to be analyzed in MD-SeeGH.

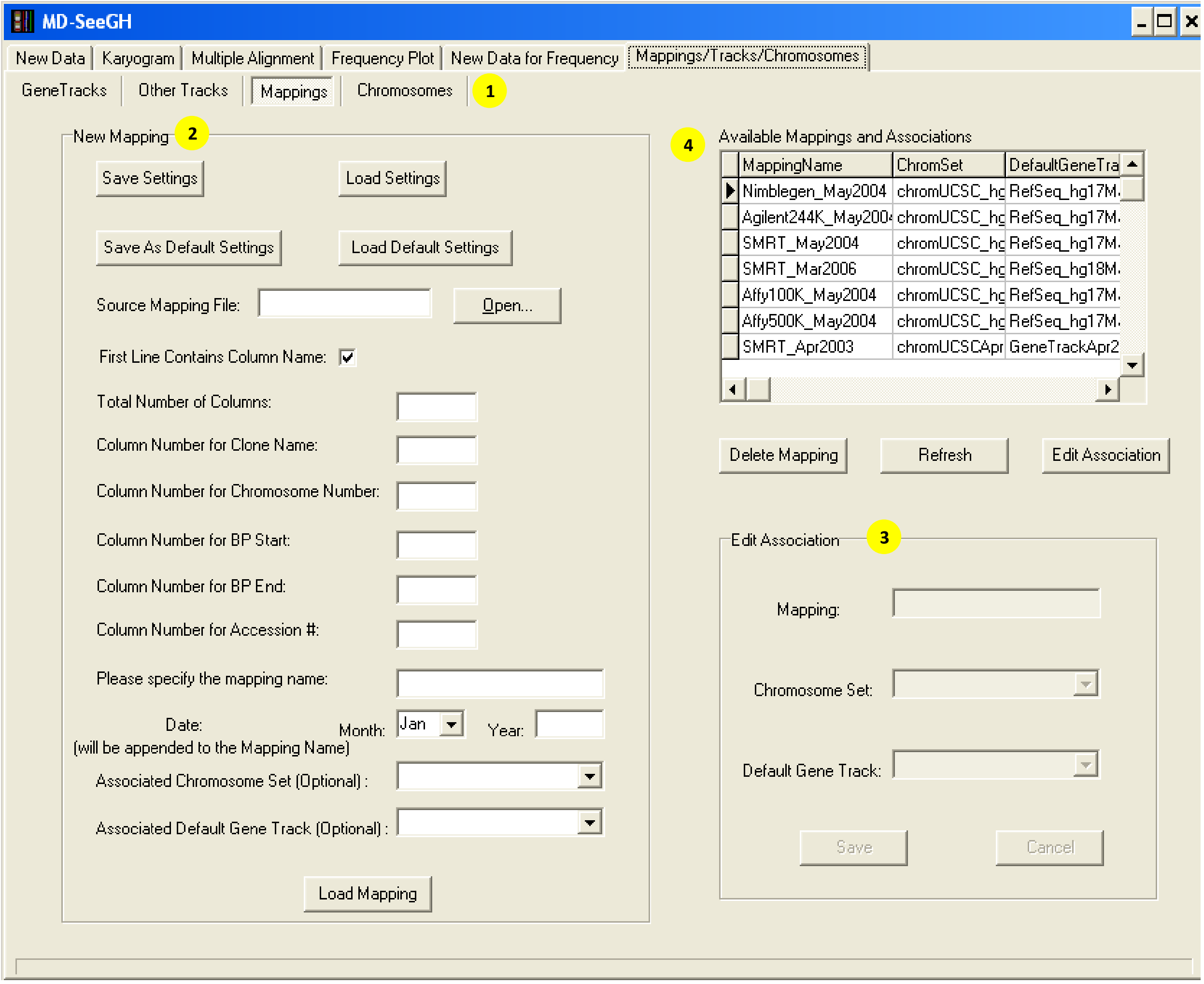


1. GeneTracks, Other Tracks, Mappings, and Chromosomes tabs where users can load new tracks, mappings, or chromosome information. Current tab selected is the Mappings tab.
2. New Mapping section where user loads a tab-delimited text file containing mapping information for a single or group of platforms. User must also enter which column contains the required data (Clone Name, Chromosome Number, Base Pair Start Position, Base Pair End Position, and Accession #.)
3. Edit Mapping section where user sets which Chromosome Set and Gene Track is associated with the Mapping file.
4. Lists all currently loaded mapping files and their associated Chromosome Sets and Default Gene Tracks. Currently, MD-SeeGH comes loaded with SMRT array (Apr03, May04, Mar06), Nimblegen (May04), Agilent 244K (May04), Affymetrix 100K and 500K (May04) mapping files.

**Figure S2. Visualization: Karyogram and Output from Segmentation Analysis**

The Karyogram allows users to analyze a singe sample using such features as segmentation probabilities, gene tracks and direct links to public databases such as NCBI and UCSC (see Figure 2 a-c below).

Figure S2a. Analysis: Genomic Plot

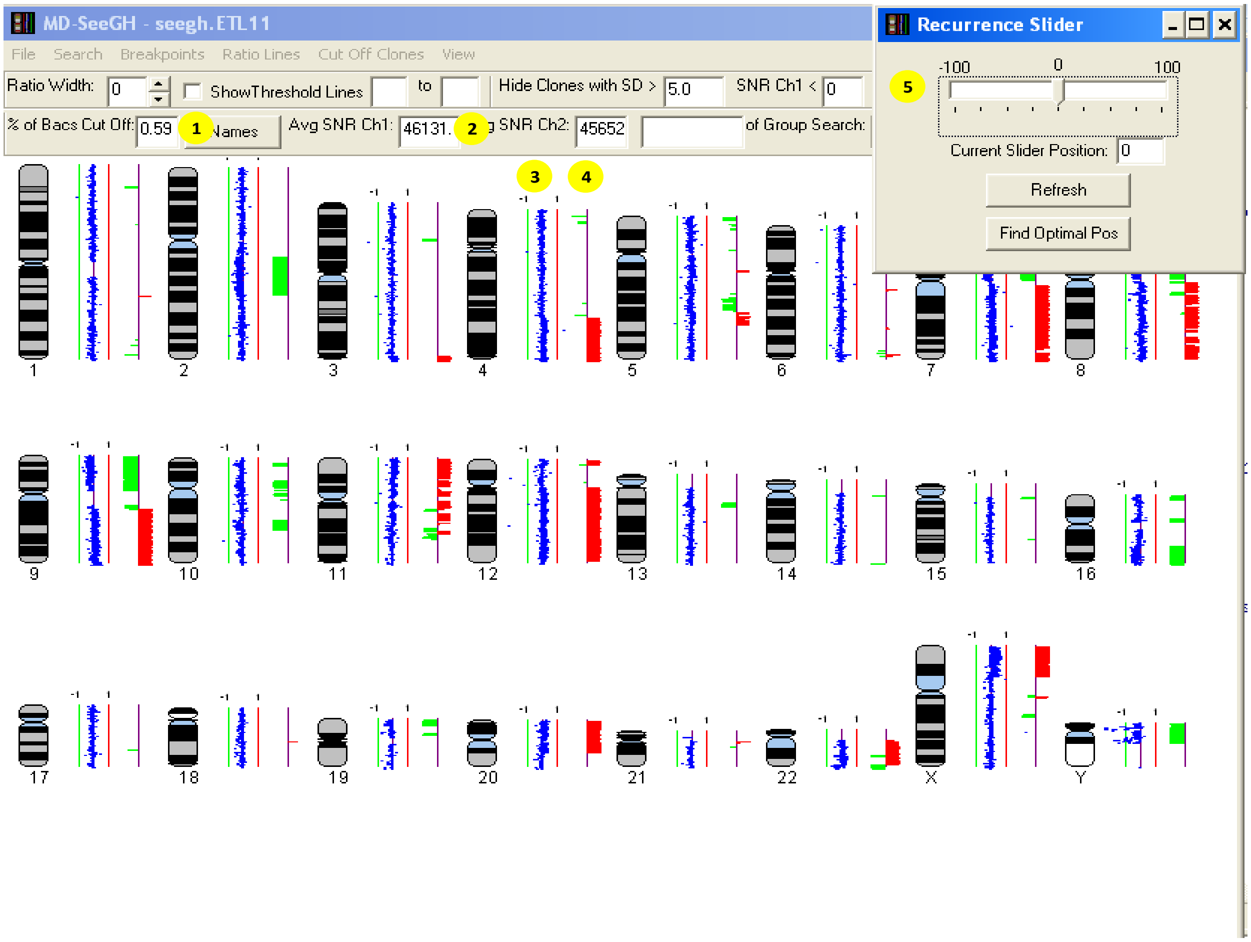


1. Displays the percentage of clones that were filtered out due to the standard deviation (SD) and signal to noise (SNR) settings.
2. Displays the average SNR for each channel across the entire sample.
3. Ratios for each array feature (e.g. a Clone) are colored blue and shown to the right of each chromosome.
4. Samples can be analyzed using our in-house segmentation algorithm CNA-HMMer (Shah, et al., 2006) and the resulting segmentation probabilities are plotted for each chromosome with amplification calls colored red and deletion calls colored green.
5. A recently described phenomenon in array CGH experimentation (regardless of array platform used) has been the identification of a recurrent artefact pattern that is independent of the copy number status (Blesa, et al., 2006)(Marioni, et al., 2007). We examined our database of greater than 2000 SMRT array experiments and selected 25 cases with the most characteristic recurrent artefact pattern. The combined profile of these cases is made available for use as a quality assessment of hybridization experiments detecting the presence of this potential artefact pattern. Users of SMRT arrays can either subtract artefact ratios manually using the slider or use the [Find Optimal Pos] button which will automatically calculate the optimal position of the slider for the maximal removal of the recurrent pattern.

Figure S2b. Analysis: Chromosome Plot

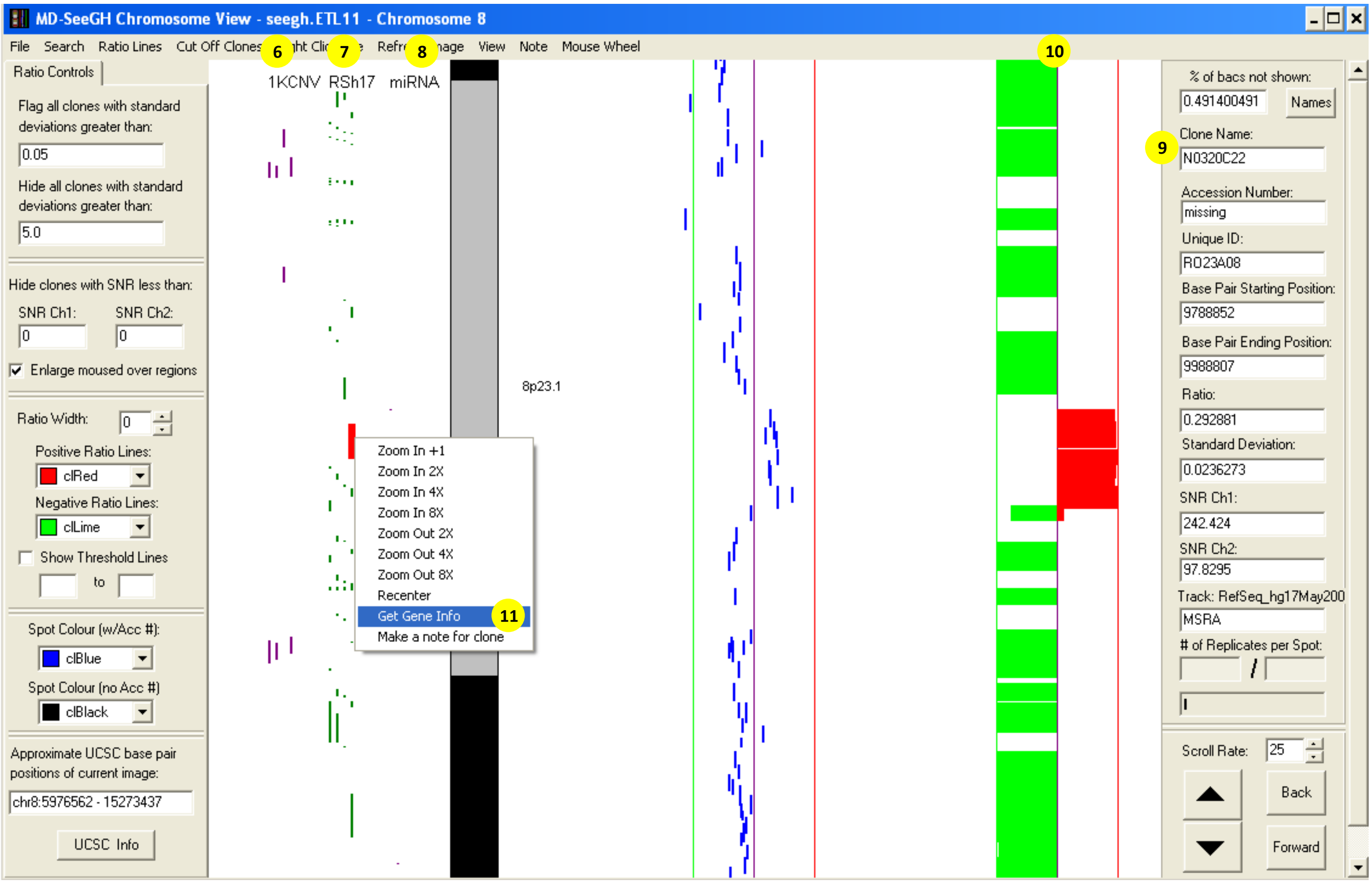


Profile can be readily compared against the following genomic maps

6. CNV Track (Wong, et al., 2007)
7. RefSeq Gene Track (data loaded from UCSC Genome Browser)
8. miRNA Track (Griffith-Jones, et al., 2006)
9. Displays the spot information (name, accession number, base pair start, base pair end, ratio, SD, SNR) when mousing over a specific feature. When mousing over a track it will display the name for the track feature as well as which track it is from.
10. Samples can be analyzed using our in-house segmentation algorithm CNA-HMMer (Shah, et al., 2006) and the resulting segmentation probabilities are plotted for each chromosome with amplification calls colored red and deletion calls colored green
11. With gene tracks, the user has the option to mouse over a gene of interest and select the 'Get Gene Info' option.

Figure S2c. Query of Gene Information: Gene Info and Public Database Portal

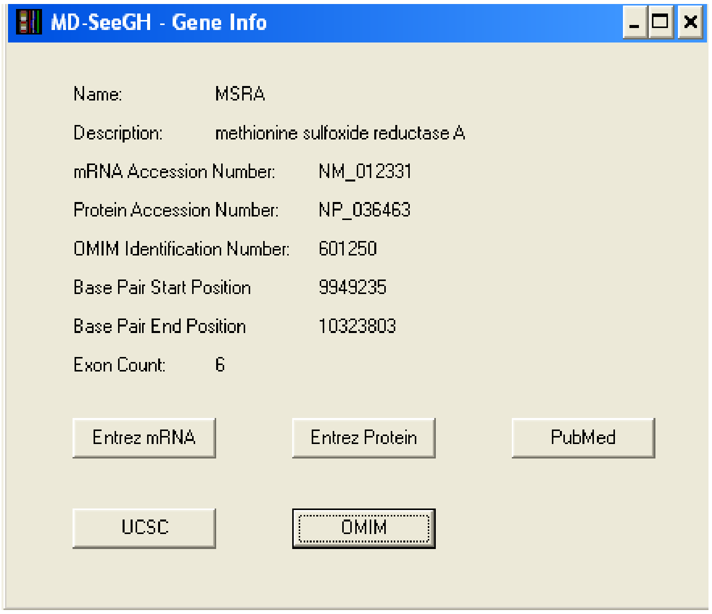


Gives detailed information on the gene and the option to query external websites for the specific gene.

Figure S2d. Query of gene information: Public Database Web Browser

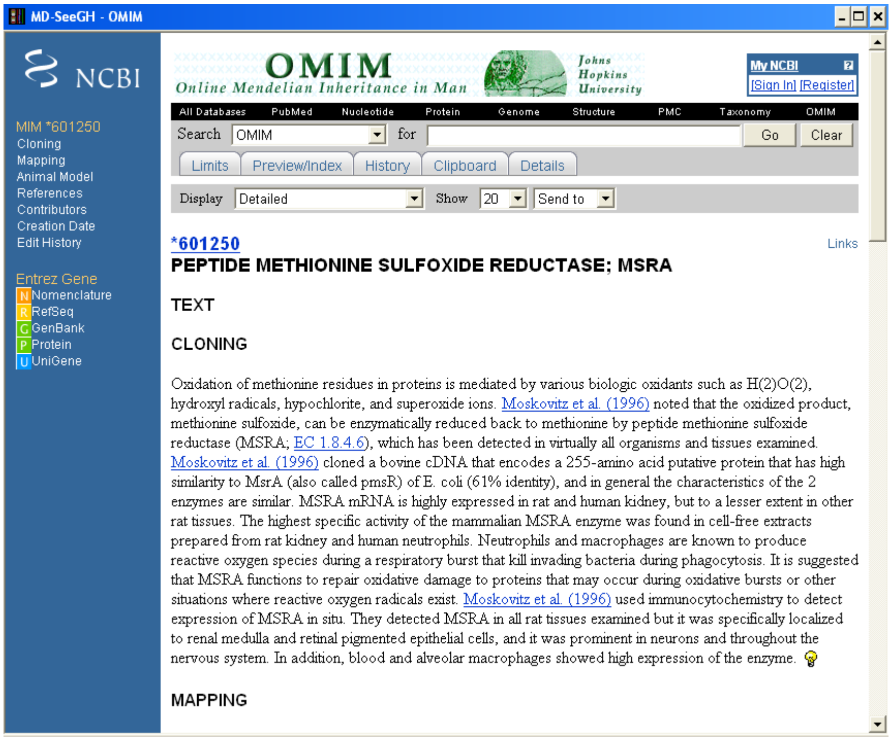


Opens when the user selects one of the corresponding buttons for Entrez mRNA, Entrez Protein, PubMed, UCSC, or OMIM

## Figure S3. Analysis: Identification and Annotation of Altered Regions

The Annotate Regions option is an analysis tool that allows you to record regions of interest (i.e. Amplifications, deletions), save them to the database, and create ISCN reports. Annotating regions can be used side by side with segmentation probabilities to verify the called regions and can also be used to compare amplification and deletions across multiple samples or create Frequency Plots for multiple samples (see Figures S3a and b below).

Figure S3a. Analysis: Karyogram Chromosome Plot

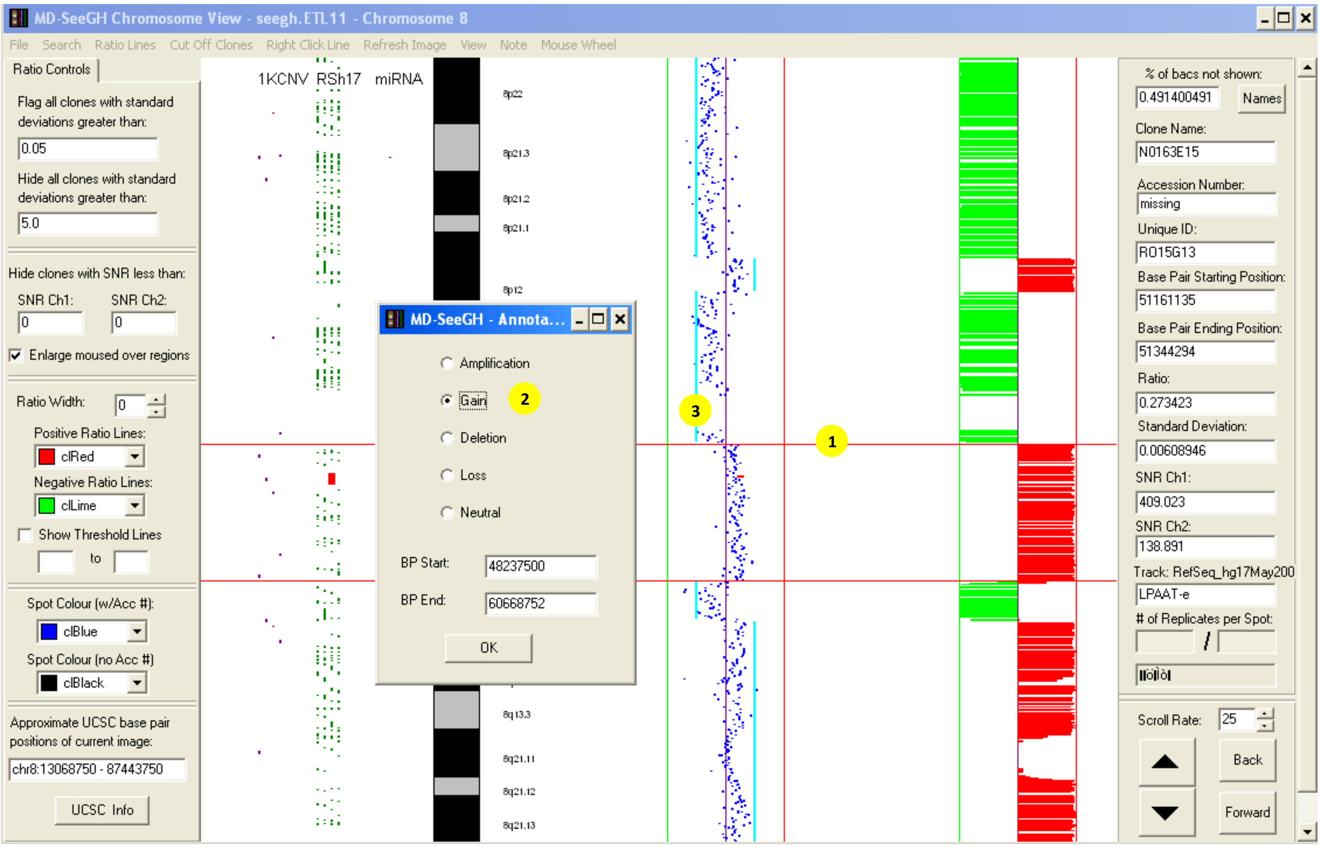


1. Right mouse clicking will draw a red line on the canvas at the mouse cursor.
2. Once two red lines have been drawn on the canvas the Annotations form will appear. This is where you can mark the region as an amplification, gain, deletion, loss or neutral region. The Annotations form also displays the base pair region of the area selected which can be edited.
3. Once annotations have been created they appear on the Chromosome View as horizontal blue lines (+1 Amplification, +0.5 Gain, -1 Deletion, -0.5 Loss, 0 Neutral).

Figure S3b.  Visualization:  Karyogram Genomic View

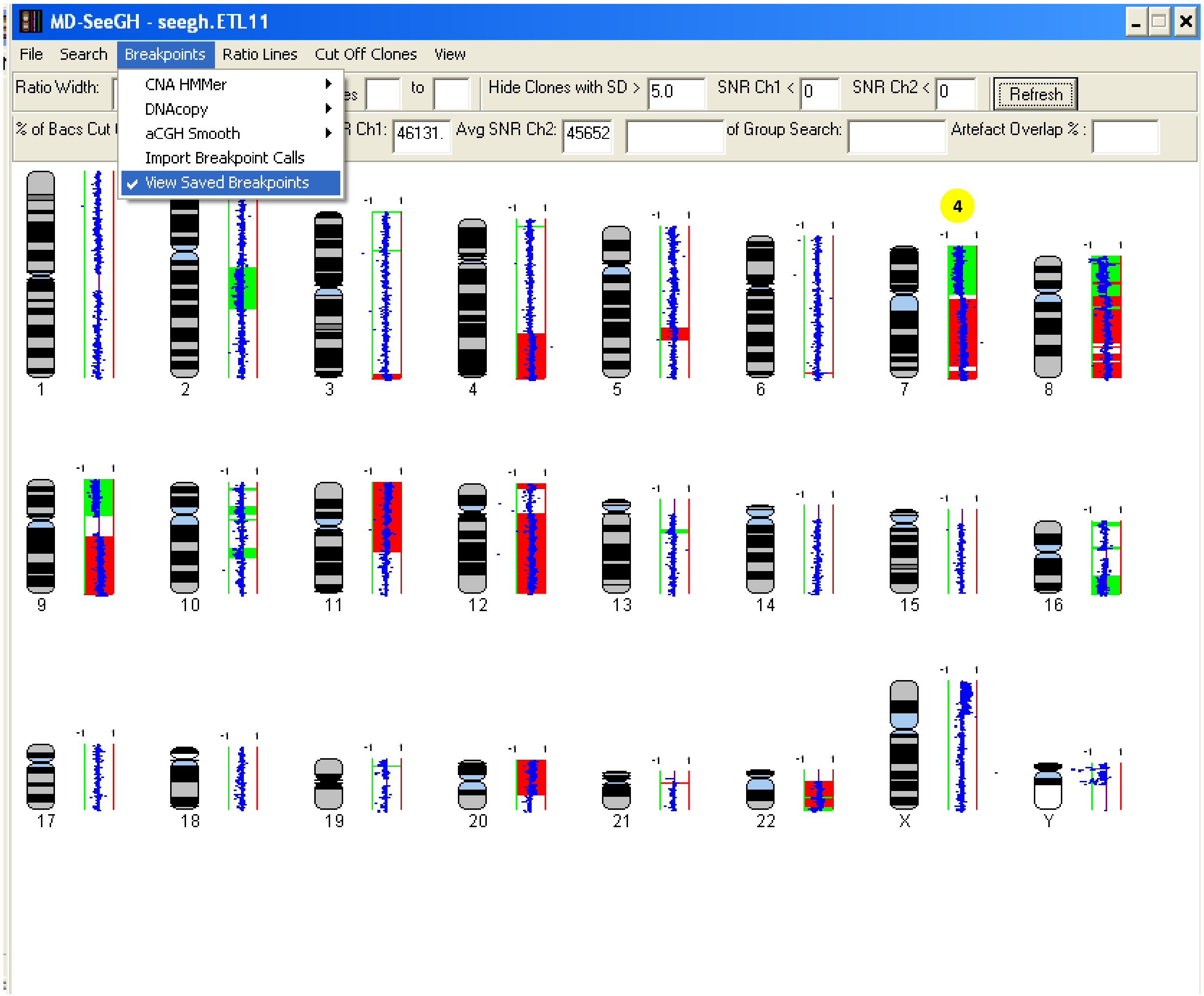


4. Once a sample's regions have been annotated and saved to the database, the user can view any previously saved calls.  This can also be done in Multiple Alignment.  Amplfications and Gains are colour-coded green.  Deletions and losses are colour coded red.

**Figure S4. Analysis: Mulitple Comparison of Different Samples from Different Array Platforms**

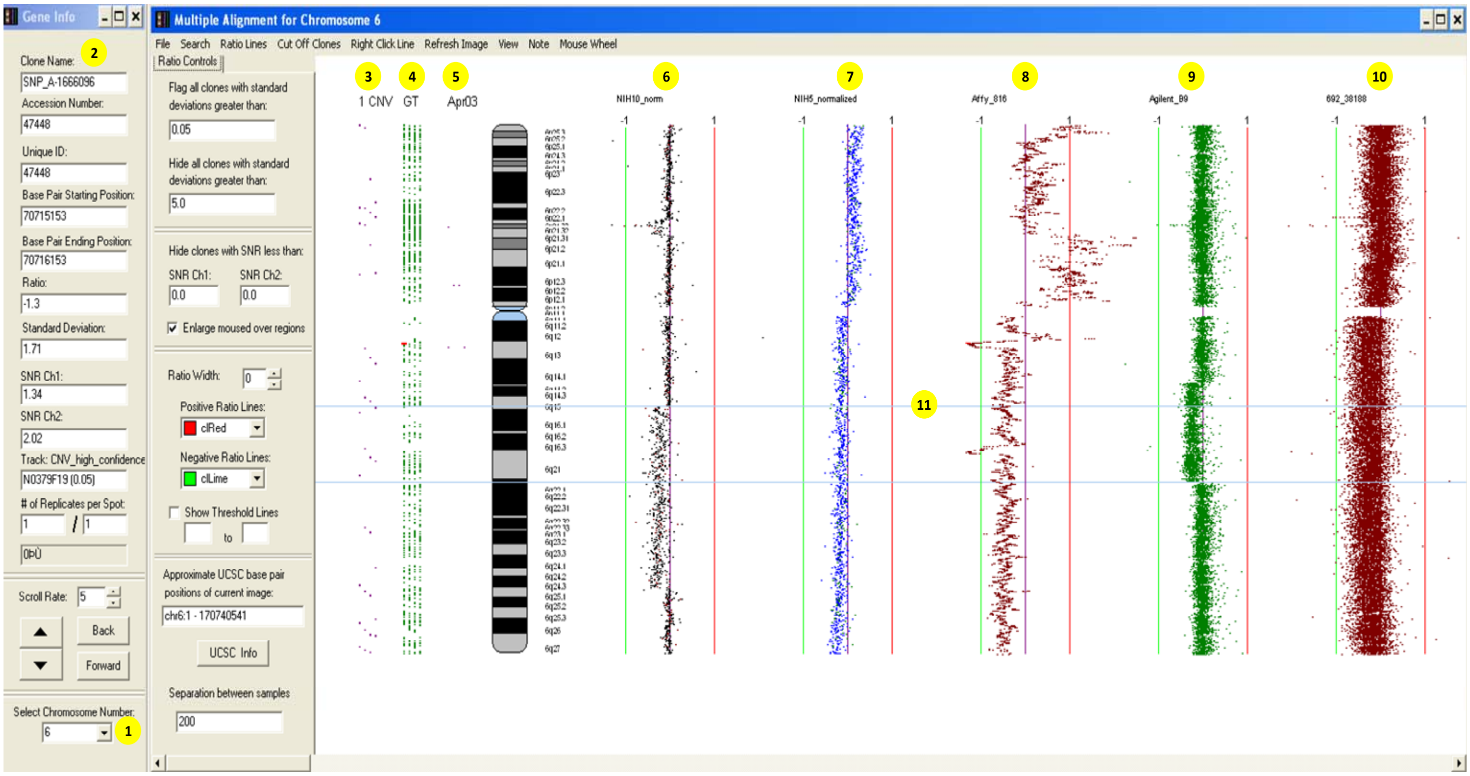


1. Allows user to change the chromosome which is currently displayed in the Multiple Alignment window.
2. Query the spot information (name, accession number, base pair start, base pair end, ratio, SD, SNR) when mousing over a specific feature. When mousing over a track it will display the name for the track feature as well as which track it is from.
3. CNV Track
4. RefSeq Gene Track
5. miRNA Track
6. SMRT Array
7. SMRT Array
8. Affymetrix 100K GeneChip
9. Agilent 244K CGH Microarray
10. Nimblegen Human Whole-Genome Array CGH
11. Right clicking with the mouse will create a blue line at the position of the mouse cursor. These can be used to mark regions of interest across all samples and export gene data or ratio data that fall between 2 selected blue lines.

Note: When aligning samples from multiple platforms a mapping file must be created containing annotation information for each platform. When creating the mapping file, the user must be certain that the annotation information for each platform is from the same genomic freeze. Note: Not all platforms have annotations for all genomic freezes.

**Figure S5. Analysis and Visualization: Comparison of the same sample (BT474 cell line) across different array platforms**

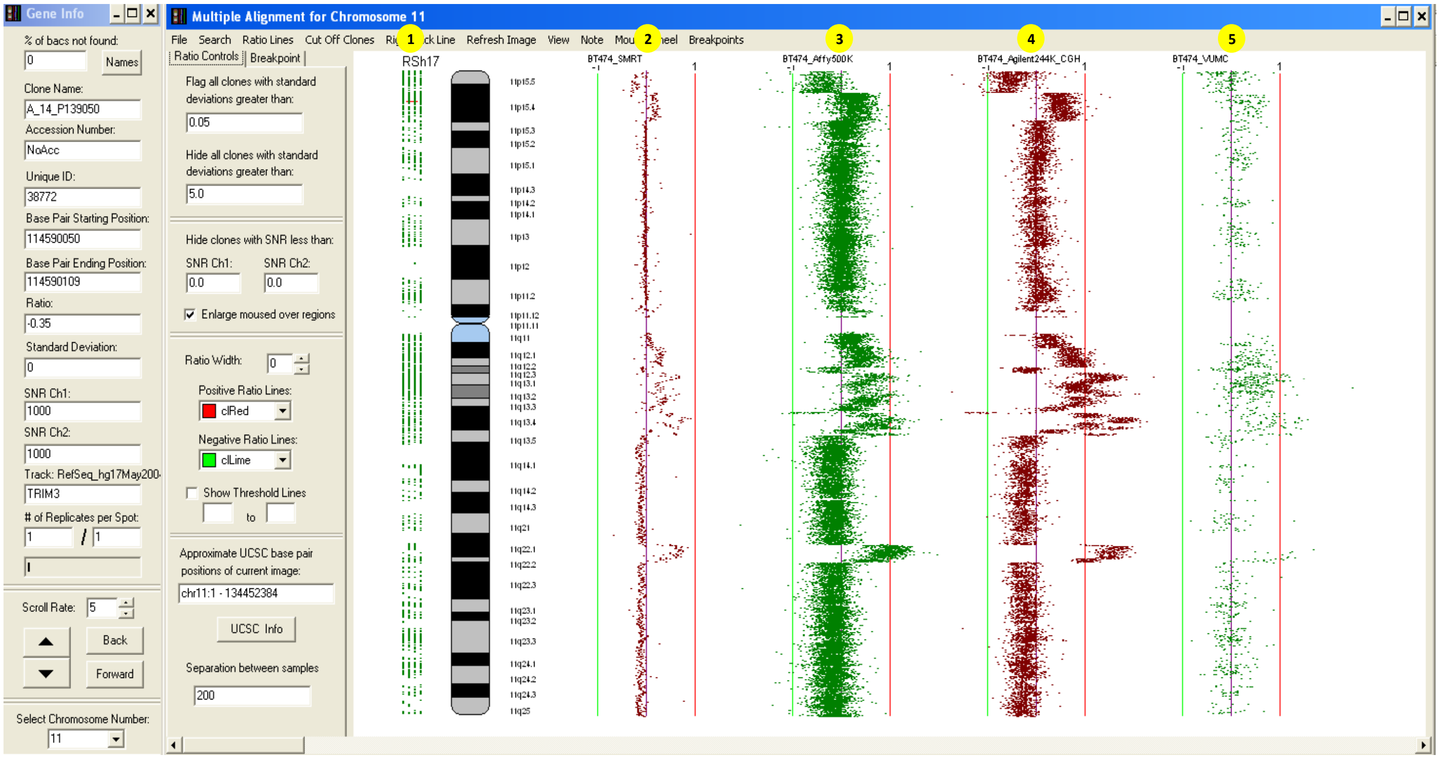

1. RefSeq Gene Track
2. SMRT array
3. Affymetrix GeneChip human mapping 500K set
4. Agilent 244A
5. VUMC MACF human 30K.

**Figure S6. Analysis of Mult-Dimensional Data: Integration of Different Types of Data**
Examples of data integration are given in Figures S6a-c.

<u>Figure S6a. Integration of Array CGH and Lymphochip cDNA Gene Expression</u>

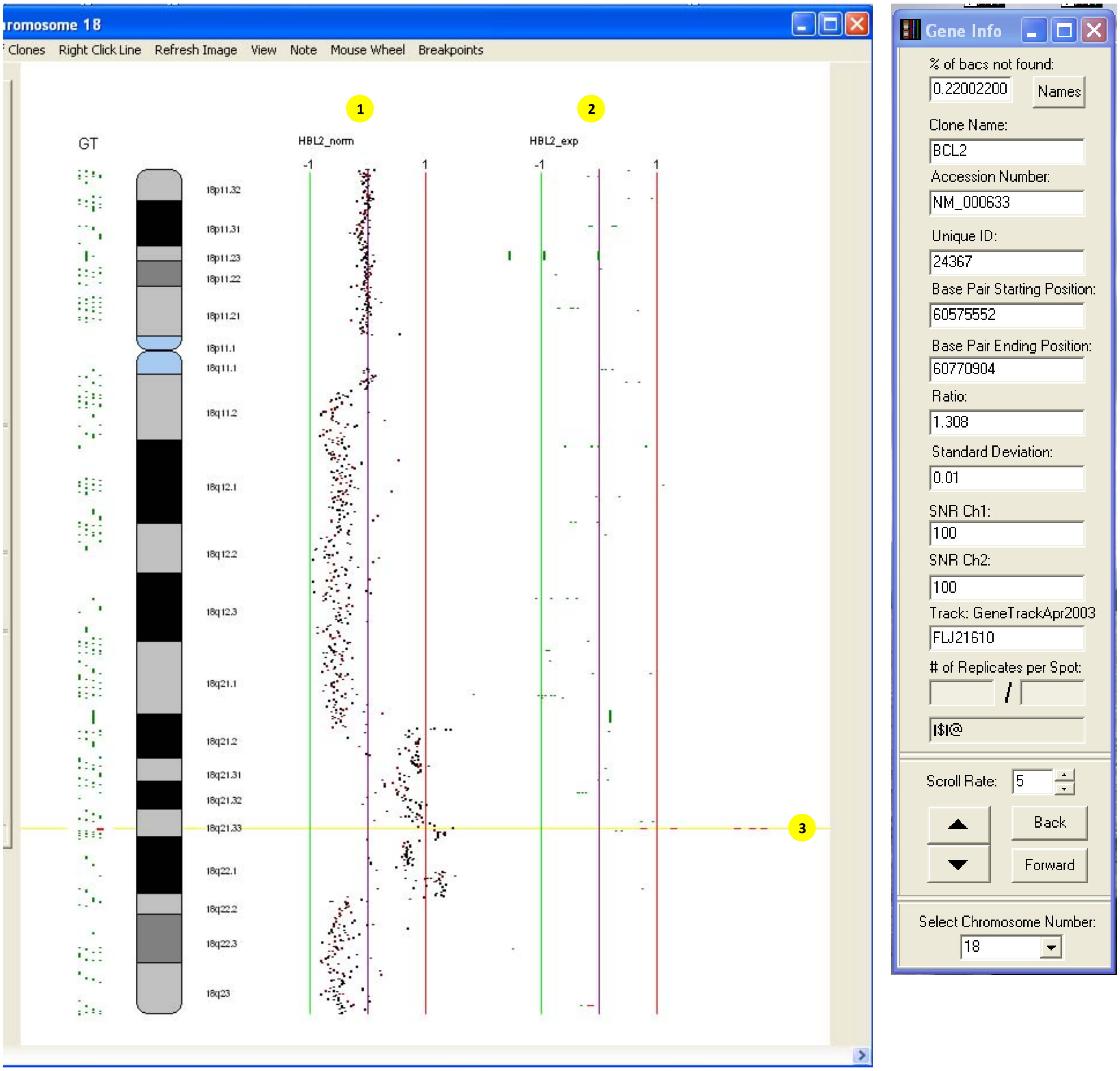


1. Array CGH profile (SMRT array)
2. cDNA expression profile (Lymphochip)
3. BCL2 gain and overexpression highlighted.

Figure S6b. Integration of epigenetic and genomic profiles

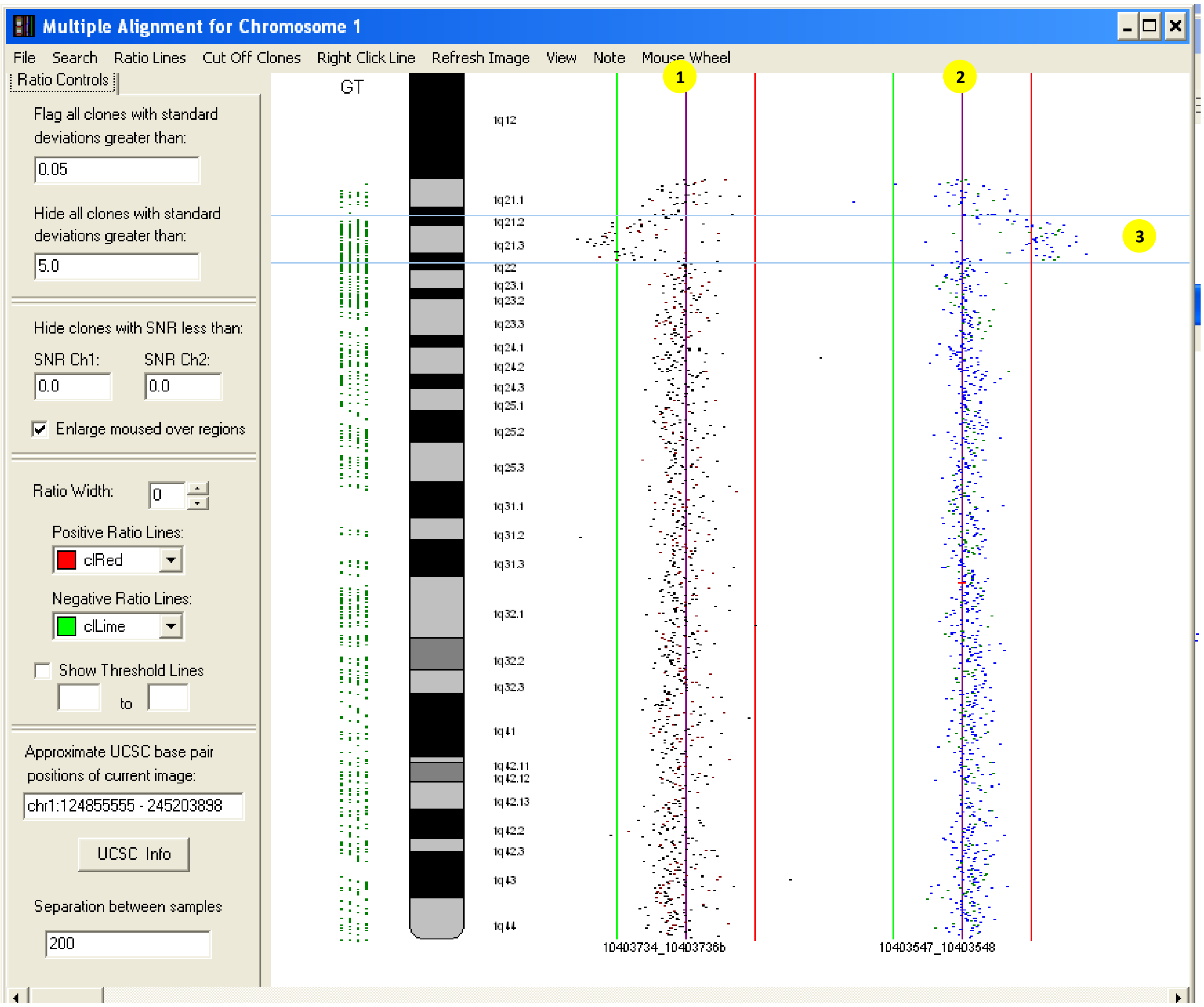


1. Methylated DNA immunoprecipitation (MeDIP) array CGH
2. Array CGH profile (SMRT array)
3. A region with both hypomethylation (left) and copy number gain (right)

Figure S6c.. Integration of copy number data in the context of SNP profile for MCF7 cells.

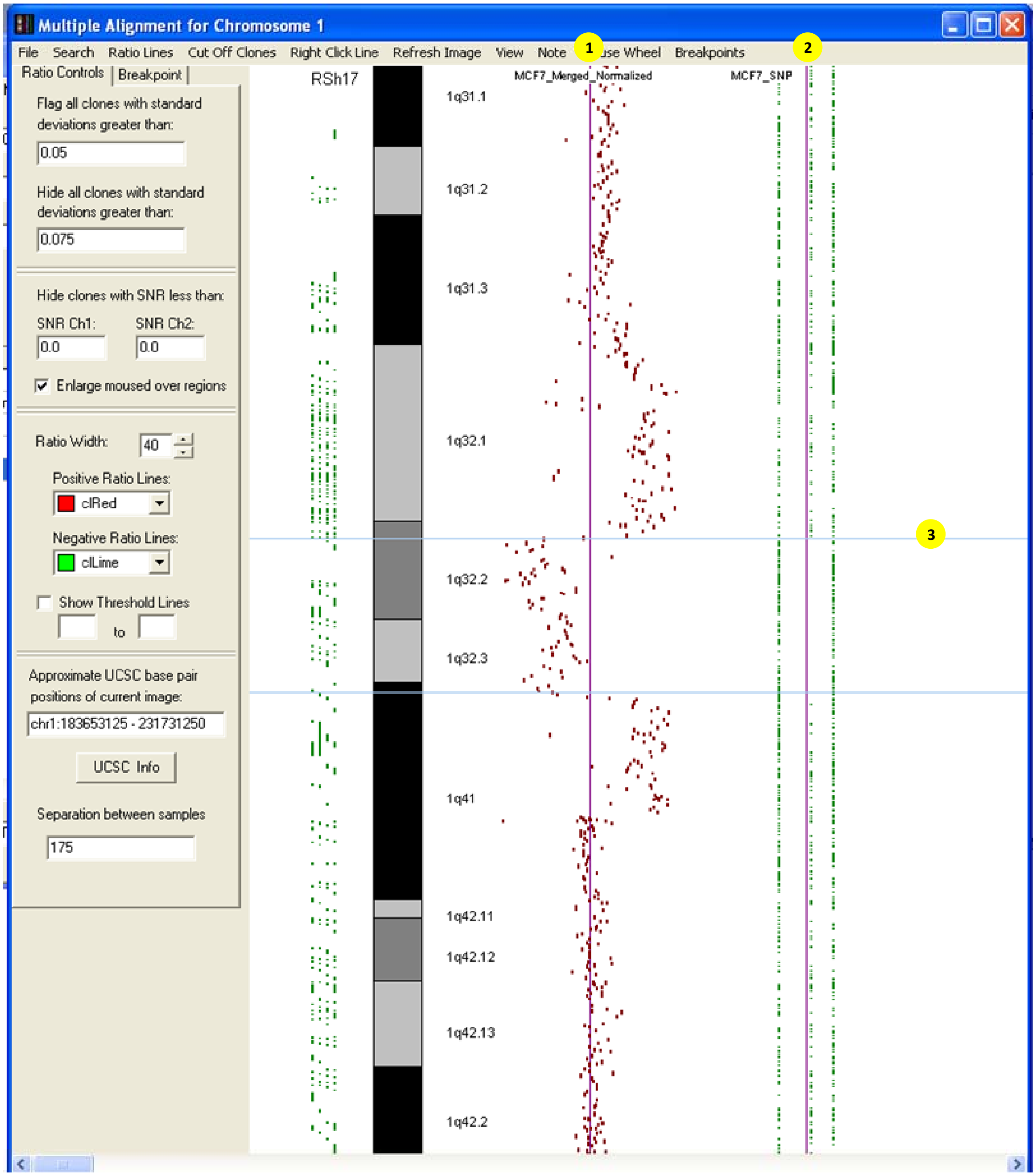


1. Array CGH profile (SMRT array)
2. Affymetrix SNP array – Homozygous AA on left, Heterozygous AB in middle, Homozygous BB on right.
3. Region between the blue lines shows a copy number loss (left) on chromosome 1 associated with LOH (right).

**Figure S7. Analysis: Heatmap for defining recurring features**

Heatmap allows the user to analyze up to 100 samples and find common regions of amplification or deletion across all or some samples (See Figures S7a-d below).

Figure S7a. Example: Heatmap alignment of 30 samples

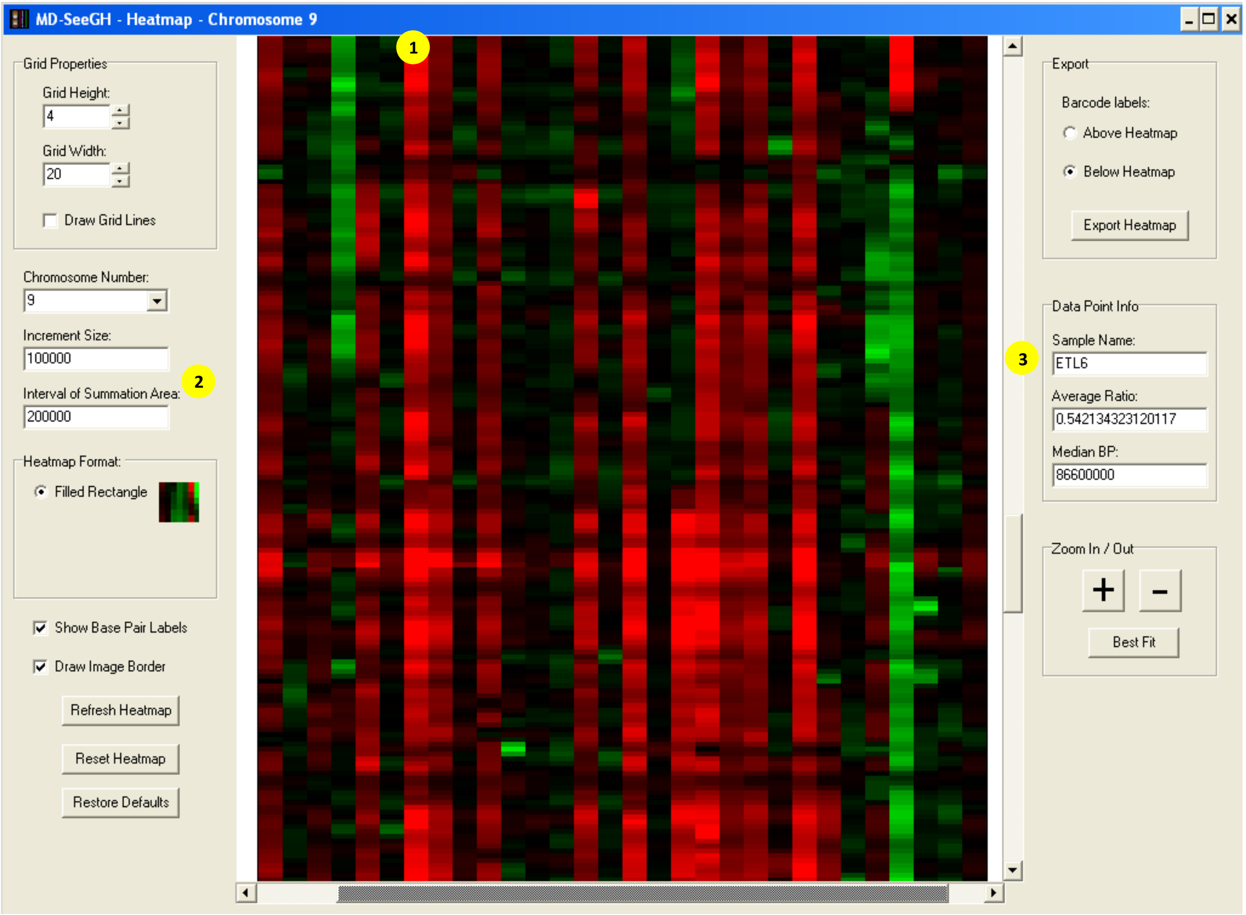


1. Each column represents a moving average heatmap of a single sample. Amplifications are shaded red and deletions are shaded green. The greater the moving average ratio the brighter the red (-) or green(+).
2. The user has the option to change how the moving average is calculated by adjusting the increment size and the interval of summation. Increment represents the base pair distance between each calculated moving average window. The interval value x 2 represents the size of the moving average window.
3. Displays the sample name, average ratio, and median base pair when mousing over the heatmap.

Figure S7b.  Analysis:  Heatmap for Defining Recurring Features

Heatmap allows the user to analyze up to 100 samples and find common regions of amplification or deletion across all or some samples. Example: Selecting regions

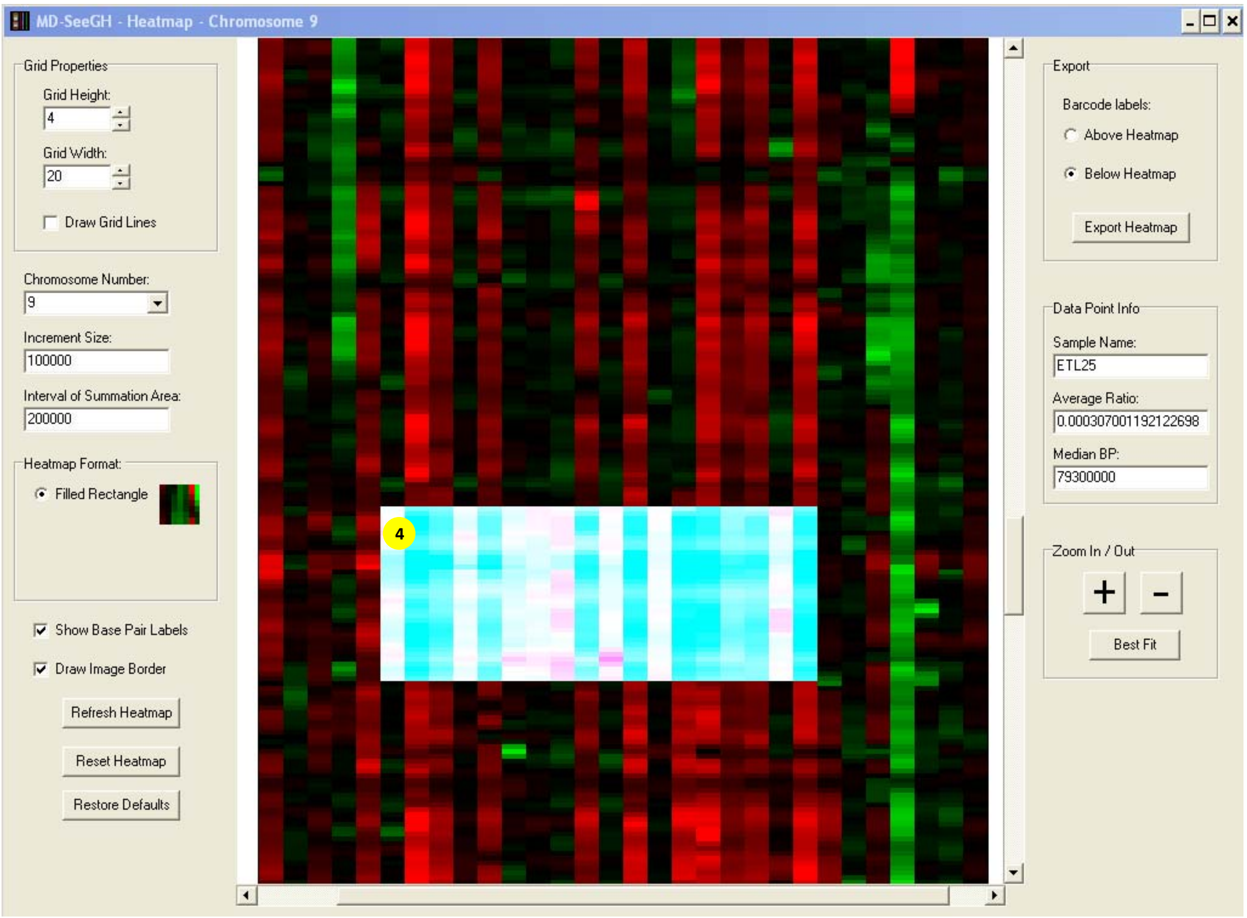


4. Click and drag of left mouse button allows you to create a box around a region of the heatmap which brings up the Multiple Alignment Parameters window.

Figure S7c.  Analysis:  Heatmap for defining recurring features

Heatmap allows the user to analyze up to 100 samples and find common regions of amplification or deletion across all or some samples.  Example: Selecting samples

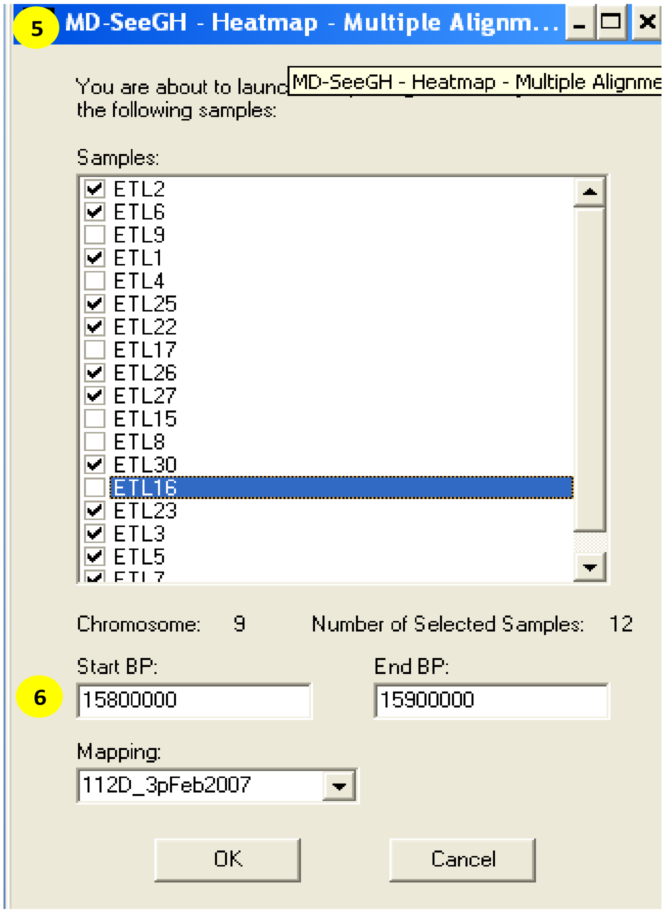


5. The Multiple Alignment Parameters window gives you the option to select samples and open them in Multiple Alignment.
6. Shows the approximate base pair region of the selected region in the heatmap.

Figure S7d.  Analysis:  Heatmap for defining recurring features

Heatmap allows the user to analyze up to 100 samples and find common regions of amplification or deletion across all or some samples.   Example: Switching from heatmap to multiple alignment

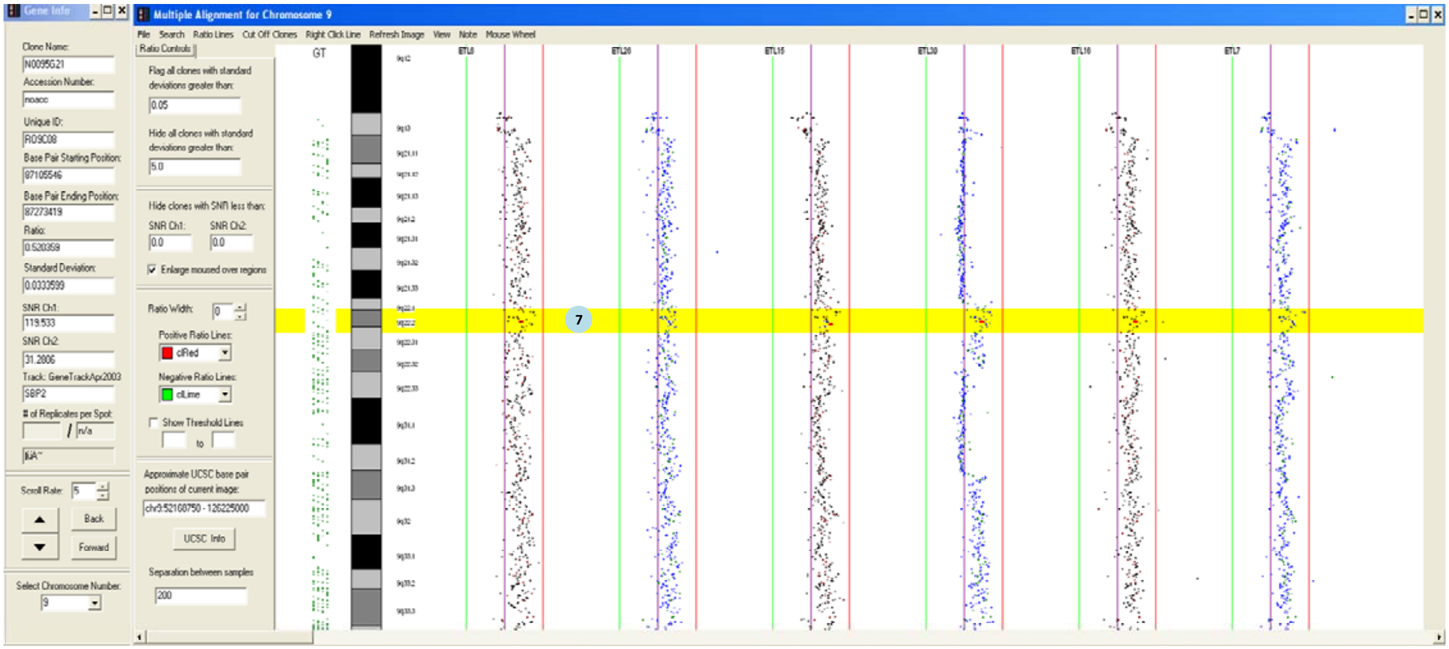

7. The region selected in the heatmap will be highlighted yellow in the Multiple Alignment View.

**Figure S8. Analysis: Frequency Plot**

The Frequency Plot can be used to analyze a group of samples and find minimal regions of amplifications or deletions. Frequency plot scoring for up to 1000 samples can be created within MD-SeeGH or created externally and loaded into MD-SeeGH. Once loaded each sample is stored in the MD-SeeGH database.

<u>Figure S8a. Genomic Frequency Plot Analysis</u>

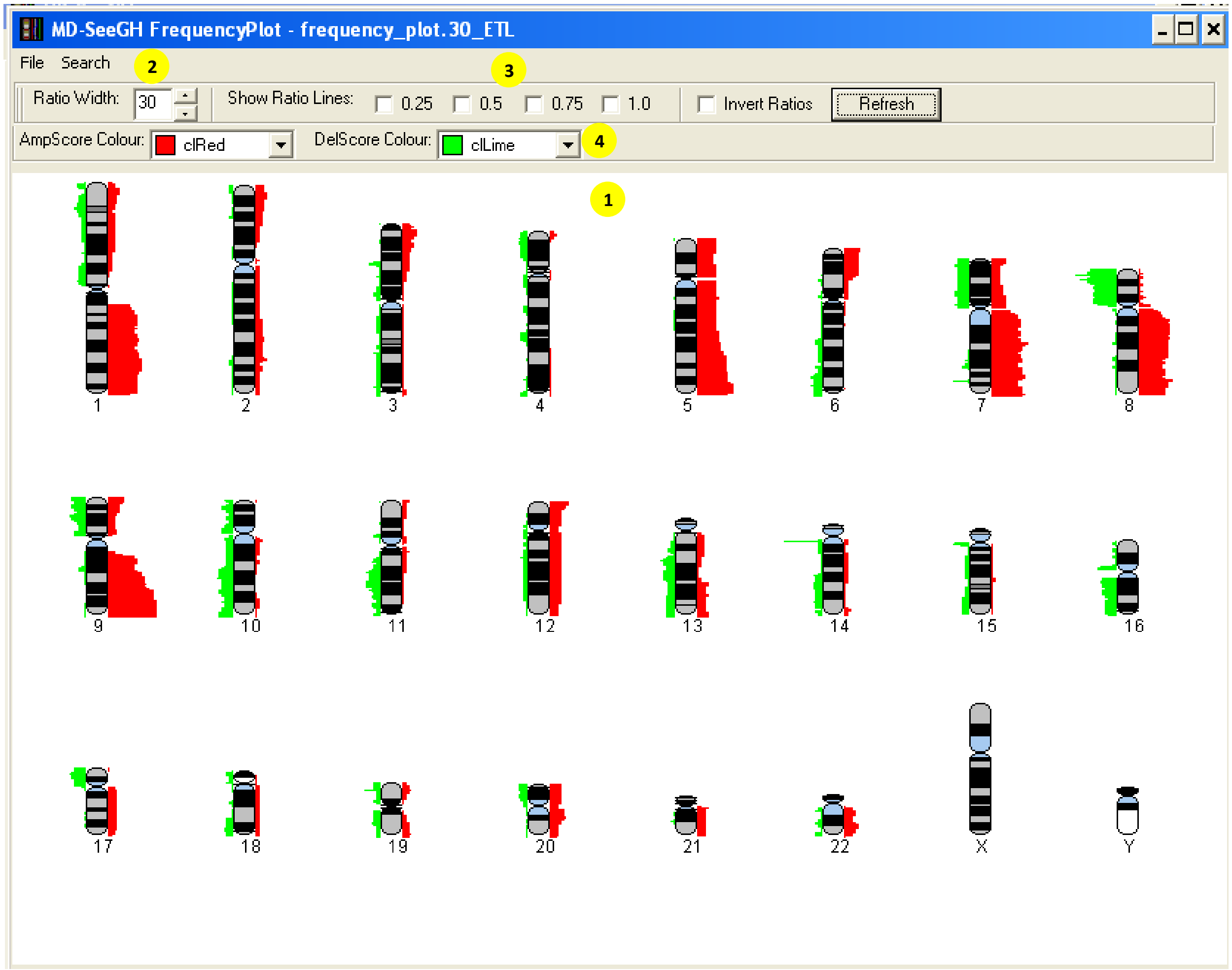


1. Frequency plot summarizing 30 ETL samples. The frequency plot shows the percentage of samples that have amplifications or deletions for each chromosomal region. Amplification scores are plotted in red and deletion scores are plotted in green. .
2. Allows you to increase or decrease the width between ratio lines.
3. Allows you to add or remove ratio lines (+/- 0.5, 1.0, 1.5, 2.0).
4. Color for amplification scores and deletion scores can be changed by the user.

<u>Figure S8b.  Chromosome Frequency Plot Analysis</u>

The Frequency Plot can be used to analyze a group of samples and find minimal regions of amplifications or deletions.  Frequency plot scoring for up to 1000 samples can be created within MD-SeeGH or created externally and loaded into MD-SeeGH.  Once loaded each sample is stored in the MD-SeeGH database.

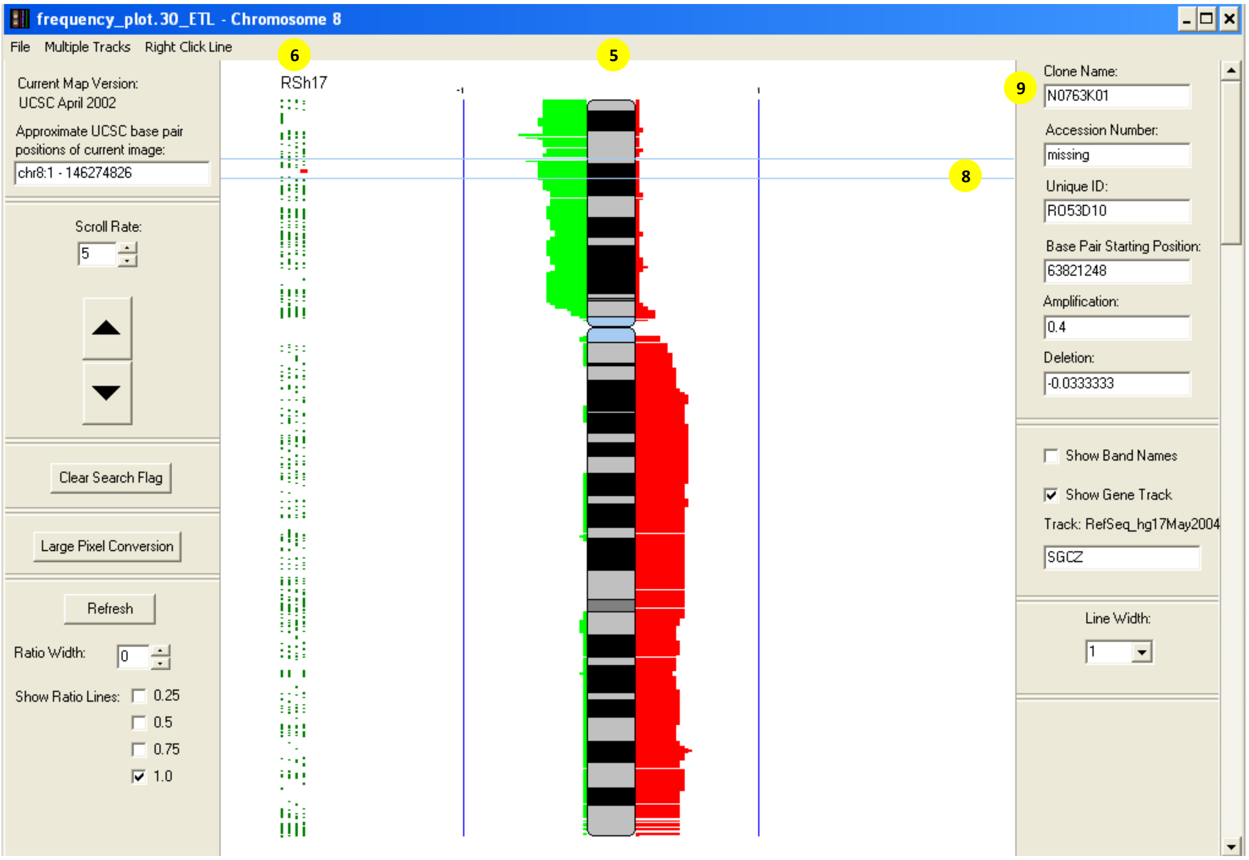


5. Frequency Plot Chromosome window has the same functionality as the Karyogram Chromosome window.
6. Gene Tracks and Additional Tracks (CNV, miRNA, etc.) can be displayed.
7. The width between ratio lines can be changed.  Ratio lines can also be added and removed.
8. Right clicking with the mouse will create a blue line at the position of the mouse cursor.  These can be used to export gene data or ratio data that fall between 2 selected blue lines.
9. Displays the feature information (name, accession number, base pair start, base pair end, amp score, del score) when mousing over the frequency plot.  When mousing over a track it will display the name for the track feature as well as which track it is from.

**Figure S9. Analysis: Group Comparison**

The Frequency Plot Comparison can be used to analyzethe similarity and differences between two groups of samples (see Figures S9a and b).

Figure S9a. Frequency Comparison between groups at the whole genome level

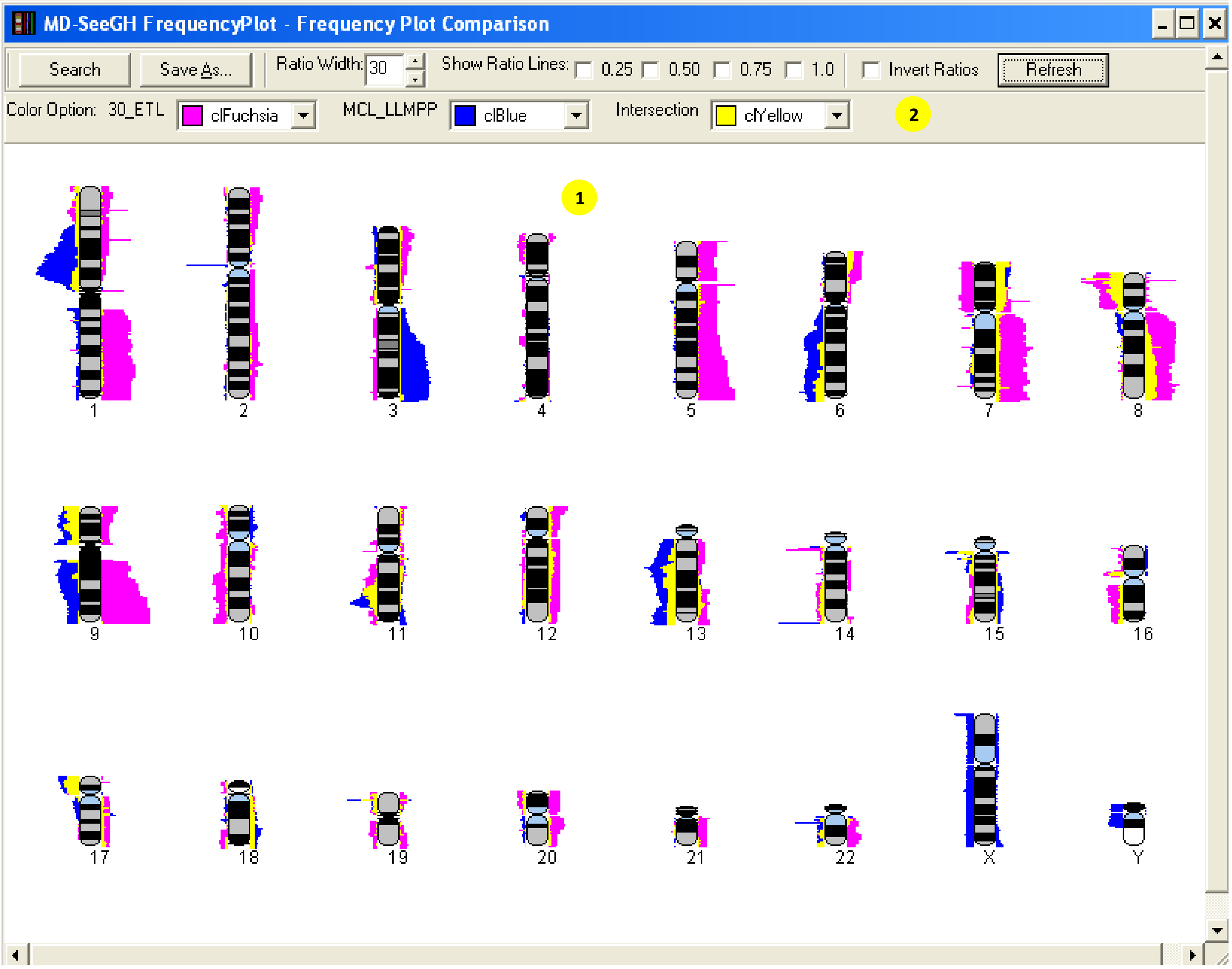


1. Frequency plot comparison of two different groups representing different disease types. Once frequency plots have been loaded/created in MD-SeeGH the user can compare two frequency plots using the overlay feature. Each group is a different color (Group 1 – Fuschia, Group 2 – Blue) and any overlapping regions are a third color (Intersection – Yellow). This is a useful feature to determine similarities and differences between groups.

2. Colors for each group and intersection can be selected by the user.

Figure S9b.  Frequency Comparison between groups at the chromosome level

The Frequency Plot Comparison can be used to analyze the similarity and differences between two groups of samples.

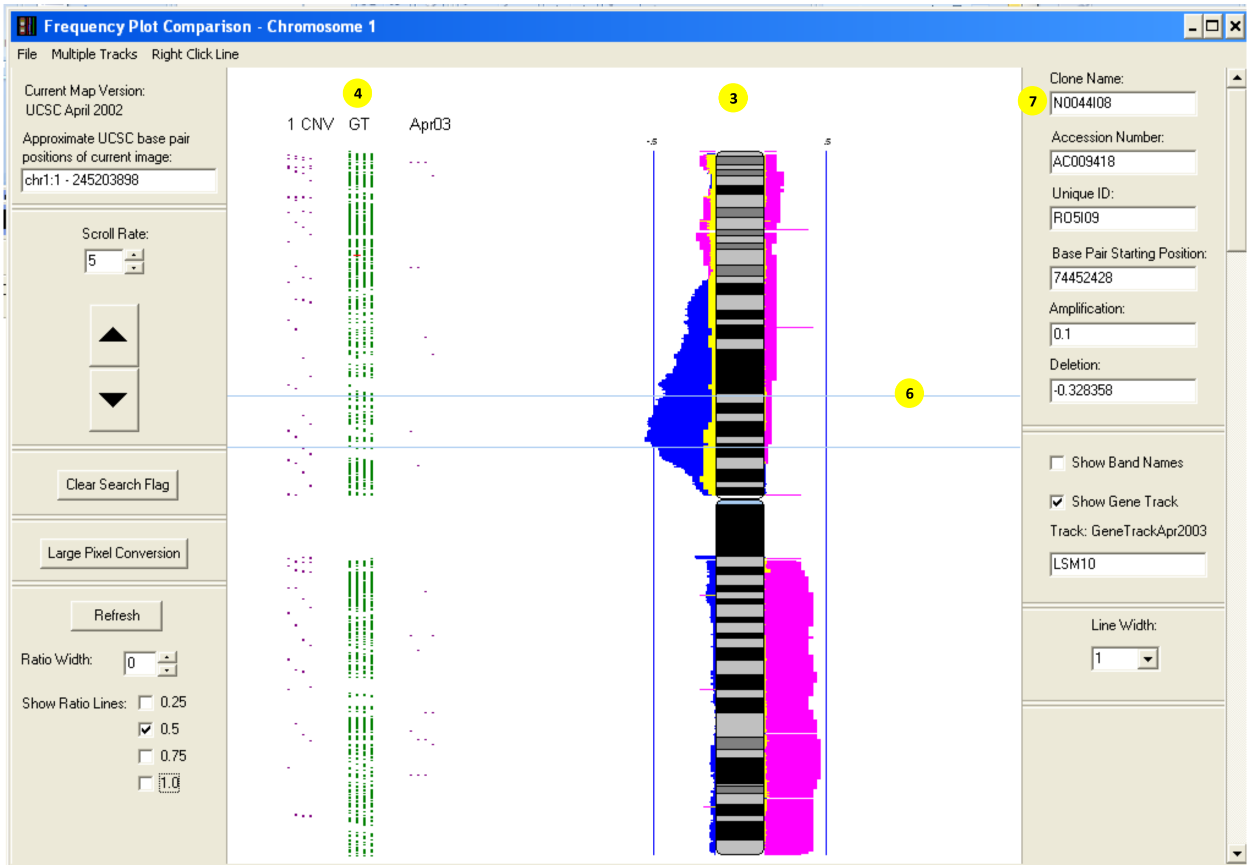


3. Frequency Plot Comparison Chromosome window has the same functionality as the Karyogram Chromosome window.
4. Gene Tracks and Additional Tracks (CNV, miRNA, etc) can be displayed.
5. The width between ratio lines can be changed.  Ratio lines can also be added and removed.
6. Right clicking with the mouse will create a blue line at the position of the mouse cursor.  These can be used to export gene data or ratio data that fall between 2 selected blue lines.
7. Displays the feature information (name, accession number, base pair start, base pair end, amp score, del score) when mousing over the frequency plot.  When mousing over a track it will display the name for the track feature as well as which track it is from.

**Figure S10. Reporting**

Figure S10a. Reporting -- Clinical report based on ISCN criteria.

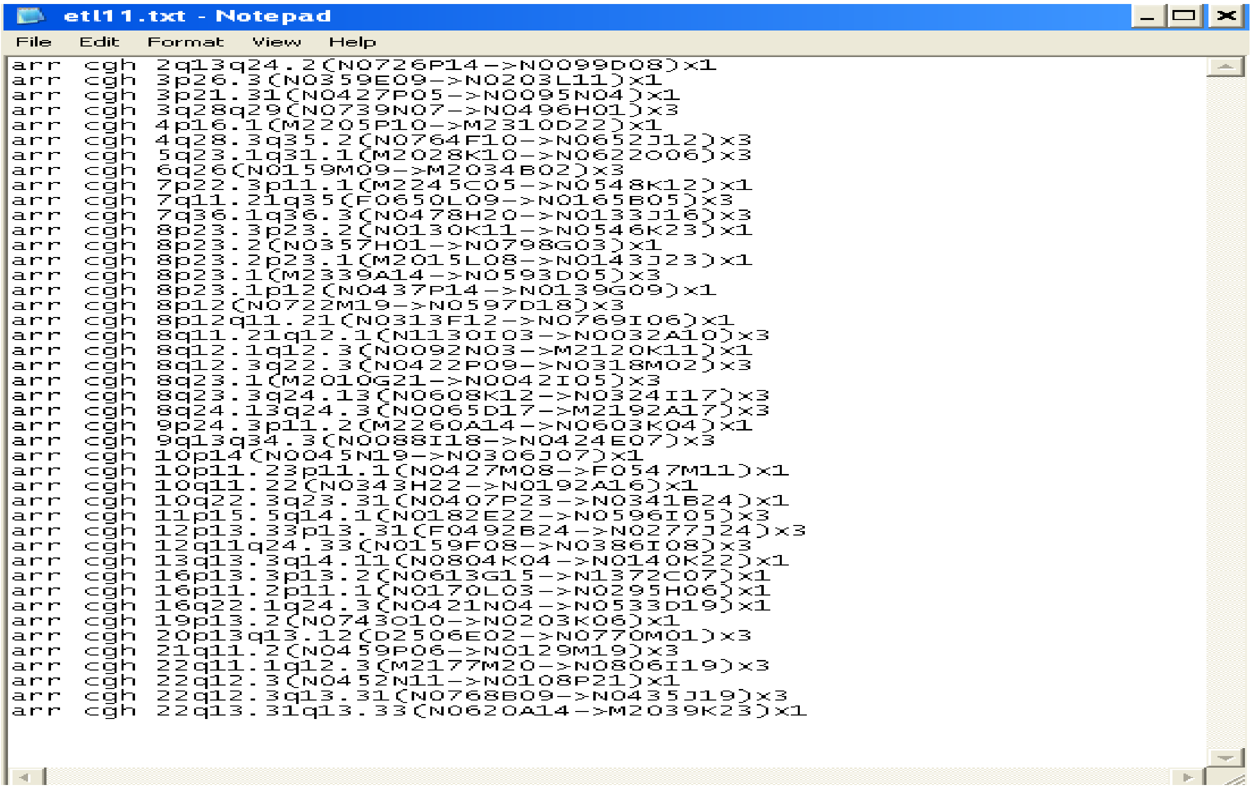

etl11.txt - Notepad

File Edit Format View Help

```
arr cgh 2q13q24.2(N0726P14->N0099D08)x1
arr cgh 3p26.3(N0359E09->N0203L11)x1
arr cgh 3p21.31(N0427P05->N0095N04)x1
arr cgh 3q28q29(N0739N07->N0496H01)x3
arr cgh 4p16.1(M2205P10->M2310D22)x1
arr cgh 4q28.3q35.2(N0764F10->N0652J12)x3
arr cgh 5q23.1q31.1(M2028K10->N0622O06)x3
arr cgh 6q26(N0159M09->M2034B02)x3
arr cgh 7p22.3p11.1(M2245C05->N0548K12)x1
arr cgh 7q11.21q35(F0650L09->N0165B05)x3
arr cgh 7q36.1q36.3(N0478H20->N0133J16)x3
arr cgh 8p23.3p23.2(N0130K11->N0546K23)x1
arr cgh 8p23.2(N0357H01->N0798G03)x1
arr cgh 8p23.2p23.1(M2015L08->N0143J23)x1
arr cgh 8p23.1(M2339A14->N0593D05)x3
arr cgh 8p23.1p12(N0437P14->N0139G09)x1
arr cgh 8p12(N0722M19->N0597D18)x3
arr cgh 8p12q11.21(N0313F12->N0769I06)x1
arr cgh 8q11.21q12.1(N1130I03->N0032A10)x3
arr cgh 8q12.1q12.3(N0092N03->M2120K11)x1
arr cgh 8q12.3q22.3(N0422P09->N0318M02)x3
arr cgh 8q23.1(M2010G21->N0042I05)x3
arr cgh 8q23.3q24.13(N0608K12->N0324I17)x3
arr cgh 8q24.13q24.3(N0065D17->M2192A17)x3
arr cgh 9p24.3p11.2(M2260A14->N0603K04)x1
arr cgh 9q13q34.3(N0088I18->N0424E07)x3
arr cgh 10p14(N0045N19->N0306J07)x1
arr cgh 10p11.23p11.1(N0427M08->F0547M11)x1
arr cgh 10q11.22(N0343H22->N0192A16)x1
arr cgh 10q22.3q23.31(N0407P23->N0341B24)x1
arr cgh 11p15.5q14.1(N0182E22->N0596I05)x3
arr cgh 12p13.33p13.31(F0492B24->N0277J24)x3
arr cgh 12q11q24.33(N0159F08->N0386I08)x3
arr cgh 13q13.3q14.11(N0804K04->N0140K22)x1
arr cgh 16p13.3p13.2(N0613G15->N1372C07)x1
arr cgh 16p11.2p11.1(N0170L03->N0295H06)x1
arr cgh 16q22.1q24.3(N0421N04->N0533D19)x1
arr cgh 19p13.2(N0743O10->N0203K06)x1
arr cgh 20p13q13.12(D2506E02->N0770M01)x3
arr cgh 21q11.2(N0459P06->N0129M19)x3
arr cgh 22q11.1q12.3(M2177M20->N0806I19)x3
arr cgh 22q12.3(N0452N11->N0108P21)x1
arr cgh 22q12.3q13.31(N0768B09->N0435J19)x3
arr cgh 22q13.31q13.33(N0620A14->M2039K23)x1
```

Regions are noted with chromosome banding position, first and last clone/feature of the region, finally whether it is an amplification or deletion. Amplifications and gains are marked as ‘x3’ while deletions and losses are marked as ‘x1’

Figure S10b. Reporting -- Publication quality images.

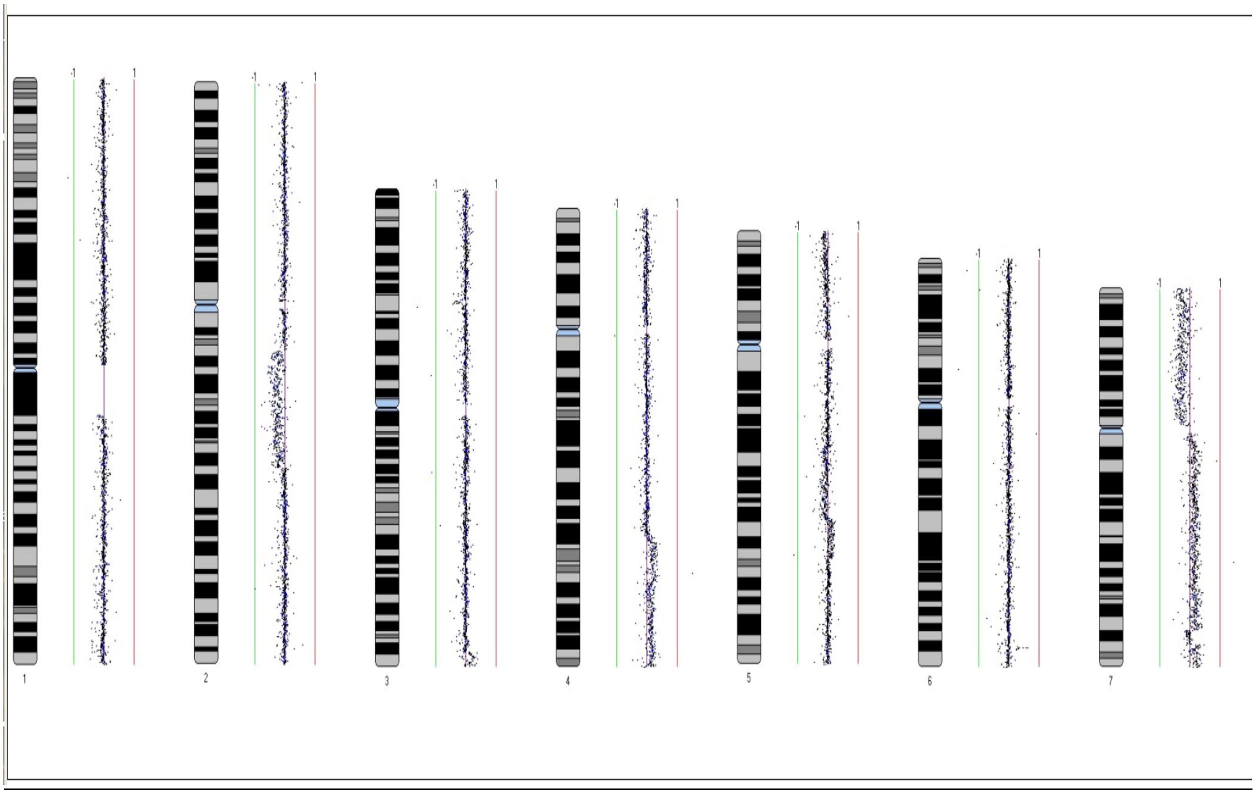


Bitmap or Jpeg images can be saved for any image in MD-SeeGH.

Figure S10c.  Reporting -- Exporting of detailed information
For example, exporting via a file containing all ratio data for a sample along with annotation information.

ETL11_data.txt

| | A | B | C | D | E | F | G | H | I | J |
|---|---|---|---|---|---|---|---|---|---|---|
| 1 | UniqueID | Clone_Name | Accession | Chromosome | BP_StartPos | BP_EndPos | Ratio | Standard_Deviation | SNR_Ch1 | SNR_Ch2 |
| 2 | RO38C24 | N0575M23 | noacc | 1 | 851036 | 1037875 | -0.0407787 | 0.0184197 | 386.03 | 151.89 |
| 3 | RO5P20 | N0054O07 | noacc | 1 | 899476 | 1049818 | -0.0773335 | 0.0257841 | 2.88919 | 3.26508 |
| 4 | RO70J19 | N0158F02 | noacc | 1 | 946127 | 1096338 | -0.0377405 | 0.0368066 | 102.957 | 53.5897 |
| 5 | RO17K23 | N0197P23 | AL713970 | 1 | 1337829 | 1419769 | -0.113766 | 0.00435422 | 73.0911 | 64.5883 |
| 6 | RO51A23 | N0730K14 | noacc | 1 | 1501818 | 1681097 | -0.0516438 | 0.0125548 | 96.4053 | 88.4982 |
| 7 | RO56H16 | N0798H13 | noacc | 1 | 1613932 | 1764429 | 0.0165799 | 0.0132778 | 148.951 | 122.513 |
| 8 | RO62O18 | N0083K22 | noacc | 1 | 1937885 | 1987762 | 0.00350849 | 0.00266079 | 75.6532 | 48.45 |
| 9 | RO67O15 | F0493G06 | noacc | 1 | 2012001 | 2175076 | -0.0364621 | 0.0560895 | 35.2322 | 14.8895 |
| 10 | RO67D03 | F0586E24 | noacc | 1 | 2118645 | 2215480 | 0.0302014 | 0.00411113 | 65.8259 | 63.9435 |
| 11 | RO61B18 | M2390G19 | noacc | 1 | 2270707 | 2327903 | -0.0114783 | 0.0682719 | 1.46956 | 0.482756 |
| 12 | RO53M01 | N0756P03 | noacc | 1 | 2321630 | 2472014 | 0.0198483 | 0.0334368 | 59.877 | 33.5507 |
| 13 | RO57B16 | N0809B17 | noacc | 1 | 2416540 | 2584920 | -0.0618942 | 0.00859825 | 89.1181 | 33.9212 |
| 14 | RO30K03 | N0427H21 | noacc | 1 | 2496932 | 2584920 | -0.0385329 | 0.00951607 | 181.804 | 85.1139 |
| 15 | RO25M12 | N0351B02 | noacc | 1 | 2640190 | 2738864 | 0.198548 | 0.00490464 | 114.156 | 46.3579 |
| 16 | RO62A13 | N0022L13 | AL590438 | 1 | 2706084 | 2856529 | -0.0468967 | 0.0418769 | 83.2737 | 260.079 |
| 17 | RO51C11 | N0730M07 | noacc | 1 | 2726375 | 2907599 | -0.0537315 | 0.0206255 | 76.2158 | 61.6112 |
| 18 | RO68K16 | N0218A10 | noacc | 1 | 2734774 | 2878457 | 0.0206796 | 0.0105359 | 3.71054 | 1.29104 |
| 19 | RO51F09 | N0736G09 | noacc | 1 | 2788808 | 2964668 | -0.0799486 | 0.00308297 | 75.3146 | 58.2927 |
| 20 | RO70P05 | N0168F09 | AL512413 | 1 | 2862721 | 3038812 | -0.0826956 | 0.0424209 | 39.0072 | 60.5618 |
| 21 | RO14G15 | N0151F10 | noacc | 1 | 2936721 | 3098099 | 0.106192 | 0.019906 | 199.129 | 49.1949 |
| 22 | RO42F24 | N0631K06 | noacc | 1 | 3103888 | 3254318 | -0.0469089 | 0.00308433 | 90.3274 | 38.2213 |
| 23 | RO68I12 | M2140D11 | noacc | 1 | 3211846 | 3332171 | -0.00804246 | 0.0229458 | 4.57269 | 1.6747 |
| 24 | RO67A14 | F0497J03 | noacc | 1 | 3239626 | 3395449 | -0.0376611 | 0.00942834 | 22.1592 | 42.8397 |
| 25 | RO50A11 | N0718D19 | noacc | 1 | 3478079 | 3671800 | -0.0334114 | 0.0199699 | 93.373 | 57.1551 |
| 26 | RO50F08 | N0727K09 | noacc | 1 | 3574073 | 3728970 | -0.03606 | 0.0303277 | 279.448 | 61.1691 |
| 27 | RO62B10 | N0118P10 | noacc | 1 | 3700081 | 3850709 | -0.130299 | 0.0345191 | 23.5059 | 12.4349 |
| 28 | RO63H12 | N0312L08 | noacc | 1 | 3742600 | 3921723 | -0.104156 | 0.0161572 | 104.646 | 29.6311 |
| 29 | RO51D10 | N0738K22 | noacc | 1 | 3848320 | 3998817 | -0.109338 | 0.0152234 | 67.8879 | 31.9561 |
| 30 | RO58B05 | N0818B16 | noacc | 1 | 3907769 | 4113516 | -0.142061 | 0.00574361 | 97.9758 | 65.4819 |
| 31 | RO68E03 | N0493P12 | noacc | 1 | 4256386 | 4442298 | 0.0454068 | 0.0543186 | 1.2993 | 0.457244 |
| 32 | RO6C02 | N0058I24 | noacc | 1 | 4283263 | 4459249 | 0.00481252 | 0.0263939 | 115.201 | 57.9016 |
| 33 | RO12P15 | N0135B10 | noacc | 1 | 4485541 | 4688400 | 0.0281615 | 0.0329961 | 187.187 | 162.202 |
| 34 | RO59J09 | M2028L18 | noacc | 1 | 4570093 | 4675282 | -0.0564379 | 0.00863788 | 70.1497 | 31.9556 |
| 35 | RO63B09 | N0245D08 | noacc | 1 | 4711874 | 4878879 | -0.0781472 | 0.0478719 | 69.8292 | 55.0302 |
| 36 | RO57N21 | N0808E08 | noacc | 1 | 4713526 | 4877166 | -0.0564527 | 0.0612091 | 24.2827 | 22.8107 |
| 37 | RO18G18 | N0230F23 | AC022922 | 1 | 5004054 | 5220021 | -0.0822568 | 0.13846 | 18.1491 | 31.5532 |
| 38 | RO14I18 | N0154H17 | AL365255 | 1 | 5096701 | 5325491 | -0.111483 | 0.139236 | 7.50204 | 2.95176 |
| 39 | RO60N14 | M2276P04 | noacc | 1 | 5398236 | 5496249 | 0.424599 | 0.0187457 | 221.875 | 83.3802 |
| 40 | RO4I24 | N0033M12 | AL356693 | 1 | 5469674 | 5542283 | -0.0139059 | 0.00973234 | 185.894 | 128.388 |
| 41 | RO41K12 | N0618E08 | noacc | 1 | 5546201 | 5719789 | 0.030897 | 0.00630757 | 5.15096 | 1.8919 |
| 42 | RO46O13 | N0668O13 | noacc | 1 | 5713634 | 5851275 | 0.00139736 | 0.0351465 | 99.7052 | 76.9315 |
| 43 | RO49B24 | N0715N23 | noacc | 1 | 5786083 | 5930945 | -0.0542654 | 0.0115391 | 54.67 | 40.8174 |
| 44 | RO29P07 | N0421D07 | noacc | 1 | 5905866 | 6024441 | -0.059601 | 0.00311288 | 253.865 | 161.074 |
| 45 | RO50E21 | N0719E21 | noacc | 1 | 6037592 | 6244322 | -0.077009 | 0.0536635 | 90.5816 | 90.7974 |
| 46 | RO6O09 | N0058A11 | AL591866 | 1 | 6086118 | 6262135 | -0.0791095 | 0.0253387 | 7.27955 | 6.95318 |
| 47 | RO4J05 | N0036K18 | noacc | 1 | 6265246 | 6415553 | -0.168972 | 0.166084 | 117.136 | 75.9464 |
| 48 | RO39O04 | N0593O14 | noacc | 1 | 6361633 | 6548503 | 0.0384964 | 0.00486135 | 172.236 | 40.6253 |
| 49 | RO63H06 | N0312B08 | AL590128 | 1 | 6433408 | 6637801 | -0.0306682 | 0.0153893 | 98.6816 | 42.7654 |
| 50 | RO34D20 | N0492B24 | noacc | 1 | 6634439 | 6867517 | -0.254057 | 0.0365067 | 1.00E+06 | 1.00E+06 |
| 51 | RO64C03 | N0334N17 | AL512330 | 1 | 6637802 | 6697306 | 0.0365569 | 0.0113892 | 102.559 | 55.4735 |
| 52 | RO16B17 | N0185M23 | noacc | 1 | 6761744 | 6949448 | 0.013938 | 0.000956058 | 5.91101 | 2.07157 |
| 53 | RO40A17 | N0600H17 | noacc | 1 | 6903255 | 7053093 | -0.034683 | 0.0304456 | 4.8721 | 1.72425 |
| 54 | [illegible] | [illegible] | [illegible] | 1 | [illegible] | [illegible] | [illegible] | [illegible] | [illegible] | [illegible] |

ETL11_data

Figure S10d. Reporting -- Describing information within a selected region.

ETL11_Chr2.txt

| | A | B | C | D | E | F | G | H | I | J | K | L | M | N |
|---|---|---|---|---|---|---|---|---|---|---|---|---|---|---|
| 1 | Sample Name: seegh.ETL11 | | | | | | | | | | | | | |
| 2 | Track Name: GeneTrackApr2003 | | | | | | | | | | | | | |
| 3 | Chromosome: 2 | | | | | | | | | | | | | |
| 4 | Gene Name | Description | mrna_acc | prot_acc | omim_id | strand | bp_start | bp_end | total_lengt | exon_count | | | | |
| 5 | BCL2L11 | BCL2-like 11 isoform 6 | NM_006538 | NP_006529 | 603827 | + | 111788137 | 111828623 | 40486 | 4 | | | | |
| 6 | ANAPC1 | anaphase-promoting complex 1 (meiotic checkpoint | NM_022662 | NP_073153 | 0 | - | 112433497 | 112548556 | 115059 | 48 | | | | |
| 7 | MERTK | c-mer proto-oncogene tyrosine kinase | NM_006343 | NP_006334 | 604705 | + | 112562999 | 112693747 | 130748 | 20 | | | | |
| 8 | FLJ14681 | hypothetical protein FLJ14681 | NM_032824 | NP_116213 | 0 | + | 112719628 | 112781445 | 61817 | 19 | | | | |
| 9 | FLJ37440 | hypothetical protein FLJ37440 | NM_153214 | NP_694946 | 0 | + | 112802776 | 112852606 | 49830 | 8 | | | | |
| 10 | LOC84524 | zinc finger protein | NM_032494 | NP_115883 | 0 | - | 112880256 | 112919465 | 39209 | 9 | | | | |
| 11 | RANBP2L1 | RAN-binding protein 2-like 1 isoform 1 | NM_005054 | NP_005045 | 602752 | - | 113032780 | 113097922 | 65142 | 23 | | | | |
| 12 | MGC46235 | hypothetical protein MGC46235 | NM_153712 | NP_714923 | 0 | + | 113146736 | 113193187 | 46451 | 7 | | | | |
| 13 | POLR1B | similar to DNA-directed RNA polymerase I (135 | NM_019014 | NP_061887 | 0 | + | 113206834 | 113242223 | 35389 | 15 | | | | |
| 14 | C2orf9 | chromosome 2 open reading frame 9 | NM_032309 | NP_115685 | 0 | + | 113248850 | 113253432 | 4582 | 4 | | | | |
| 15 | SLC20A1 | solute carrier family 20 (phosphate | NM_005415 | NP_005406 | 137570 | + | 113310422 | 113328217 | 17795 | 11 | | | | |
| 16 | FLJ40629 | hypothetical protein FLJ40629 | NM_152515 | NP_689728 | 0 | - | 113402258 | 113429009 | 26751 | 10 | | | | |
| 17 | IL1A | interleukin 1, alpha proprotein | NM_000575 | NP_000566 | 147760 | - | 113438307 | 113449786 | 11479 | 7 | | | | |
| 18 | IL1B | interleukin 1, beta proprotein | NM_000576 | NP_000567 | 147720 | - | 113494151 | 113501171 | 7020 | 7 | | | | |
| 19 | IL1F7 | interleukin 1 family, member 7 isoform 1 | NM_014439 | NP_055254 | 605510 | + | 113577362 | 113583271 | 5909 | 5 | | | | |
| 20 | IL1F9 | interleukin 1 family, member 9 | NM_019618 | NP_062564 | 605542 | + | 113642420 | 113650042 | 7622 | 5 | | | | |
| 21 | IL1F6 | interleukin 1 family, member 6 (epsilon) | NM_014440 | NP_055255 | 605509 | + | 113670263 | 113672436 | 2173 | 4 | | | | |
| 22 | IL1F8 | interleukin 1 family, member 8 isoform 1 | NM_014438 | NP_055253 | 605508 | - | 113686482 | 113717255 | 30773 | 6 | | | | |
| 23 | IL1F5 | interleukin 1 family, member 5 | NM_173170 | NP_775262 | 605507 | + | 113723029 | 113729133 | 6104 | 5 | | | | |
| 24 | IL1F10 | interleukin 1 family, member 10 | NM_173161 | NP_775184 | 0 | + | 113732361 | 113740240 | 7879 | 5 | | | | |
| 25 | IL1RN | interleukin 1 receptor antagonist isoform 3 | NM_000577 | NP_000568 | 147679 | + | 113782284 | 113798407 | 16123 | 5 | | | | |
| 26 | TIC | SEC7 homolog | NM_012455 | NP_036587 | 0 | + | 113846784 | 113867300 | 20516 | 16 | | | | |
| 27 | PAX8 | paired box gene 8 isoform PAX8C | NM_013952 | NP_039246 | 167415 | - | 113882907 | 113943302 | 60395 | 12 | | | | |
| 28 | FOXD4L1 | forkhead box D4-like 1 | NM_012184 | NP_036316 | 0 | + | 114163626 | 114164924 | 1298 | 1 | | | | |
| 29 | MGC13005 | hypothetical protein MGC13005 | NM_032685 | NP_116074 | 0 | - | 114263424 | 114264151 | 727 | 1 | | | | |
| 30 | RABL2A | RAB, member of RAS oncogene family-like 2A | NM_007082 | NP_009013 | 605412 | + | 114291697 | 114307780 | 16083 | 10 | | | | |
| 31 | FLJ22004 | hypothetical protein FLJ22004 | NM_025181 | NP_079457 | 0 | - | 114378746 | 114421214 | 42468 | 16 | | | | |
| 32 | ACTR3 | ARP3 actin-related protein 3 homolog | NM_005721 | NP_005712 | 604222 | + | 114554455 | 114622536 | 68081 | 12 | | | | |
| 33 | DPRP3 | dipeptidyl peptidase IV-related protein 3 | NM_020868 | NP_065919 | 0 | + | 115106712 | 116508750 | 1402038 | 26 | | | | |
| 34 | | | | | | | | | | | | | | |
| 35 | | | | | | | | | | | | | | |
| 36 | | | | | | | | | | | | | | |
| 37 | Track Name: CNV_high_confidence_Wong_et_alApr2003Apr2003 | | | | | | | | | | | | | |
| 38 | Chromosome: 2 | | | | | | | | | | | | | |
| 39 | Name | Chromosome | BP_Start | BP_End | | | | | | | | | | |
| 40 | N0068E19 (0.05) | 112104518 | 112310175 | | | | | | | | | | | |
| 41 | N0793H14 (0.12) | 113796244 | 113895127 | | | | | | | | | | | |
| 42 | | | | | | | | | | | | | | |
| 43 | | | | | | | | | | | | | | |
| 44 | | | | | | | | | | | | | | |
| 45 | | | | | | | | | | | | | | |
| 46 | | | | | | | | | | | | | | |
| 47 | | | | | | | | | | | | | | |
| 48 | | | | | | | | | | | | | | |
| 49 | | | | | | | | | | | | | | |
| 50 | | | | | | | | | | | | | | |
| 51 | | | | | | | | | | | | | | |
| 52 | | | | | | | | | | | | | | |
| 53 | | | | | | | | | | | | | | |

ETL11_Chr2

For example, exported file contains all genes and CNVs that fall in between a user defined region (blue lines).

**Figure S11. MD-SeeGH Visualization Hierarchy**

Single Sample
- Whole genome (low res)
  - selectable ratio scale lines
  - invert ratios possible
  - cutoff clones colored or masked
  - independent x-axis zoom
  - color scheme selectable
  - add altered regions as overlay
  - add probabilities of alteration beside each chromosome
- Individual chromosome (high res)
  - all additional tracks viewable
  - selectable ratio scale lines
  - invert ratios possible
  - cutoff clones colored or masked
  - independent x-axis zoom
  - y-axis zoom on mouse click
  - color scheme selectable
  - add altered regions as overlay
  - add probabilities of alteration beside each chromosome
  - blue line annotation available with associated exports

Multiple Alignment
- No whole genome view
- Individual Chromosome (up to 50 samples, high res)
  - all additional tracks viewable
  - selectable ratio scale lines
  - invert ratios possible
  - cutoff clones colored or masked
  - independent x-axis zoom
  - y-axis zoom on mouse click
  - color scheme selectable
  - add altered regions as overlay
- Heatmap (up to 100 samples)
  - independent x-axis scale
  - independent y-axis scale
  - color scheme selectable

Frequency Plot and Frequency Plot comparison(up to 1000 samples)
- Whole genome (low res)
  - selectable ratio scale lines
  - independent x-axis zoom
  - color scheme selectable
- Individual Chromosome
  - all additional tracks viewable
  - selectable ratio scale lines
  - independent x-axis zoom
  - y-axis zoom on mouse click
  - blue line annotation available with associated exports
  - color scheme selectable
